# A CRISPR Dropout Screen Identifies Genetic Vulnerabilities and Therapeutic Targets in Acute Myeloid Leukemia

**DOI:** 10.1016/j.celrep.2016.09.079

**Published:** 2016-10-18

**Authors:** Konstantinos Tzelepis, Hiroko Koike-Yusa, Etienne De Braekeleer, Yilong Li, Emmanouil Metzakopian, Oliver M. Dovey, Annalisa Mupo, Vera Grinkevich, Meng Li, Milena Mazan, Malgorzata Gozdecka, Shuhei Ohnishi, Jonathan Cooper, Miten Patel, Thomas McKerrell, Bin Chen, Ana Filipa Domingues, Paolo Gallipoli, Sarah Teichmann, Hannes Ponstingl, Ultan McDermott, Julio Saez-Rodriguez, Brian J.P. Huntly, Francesco Iorio, Cristina Pina, George S. Vassiliou, Kosuke Yusa

**Affiliations:** 1 Wellcome Trust Sanger Institute, Hinxton, Cambridge CB10 1SA, UK; 2 European Molecular Biology Laboratory, European Bioinformatics Institute, Hinxton, Cambridge CB10 1SD, UK; 3 Department of Haematology, NHS Blood and Transplant, Cambridge Biomedical Campus, University of Cambridge, Cambridge CB2 0PT, UK; 4 Department of Haematology, Cambridge University Hospitals NHS Trust, Cambridge CB2 0QQ, UK; 5 Wellcome Trust-MRC Stem Cell Institute, Cambridge Biomedical Campus, University of Cambridge, Cambridge CB2 0XY, UK; 6 Faculty of Medicine, Joint Research Center for Computational Biomedicine, RWTH Aachen, 52074 Aachen, Germany

**Keywords:** CRISPR, genetic screen, genetic vulnerability, acute myeloid leukemia, AML, KAT2A, MB-3

## Abstract

Acute myeloid leukemia (AML) is an aggressive cancer with a poor prognosis, for which mainstream treatments have not changed for decades. To identify additional therapeutic targets in AML, we optimize a genome-wide clustered regularly interspaced short palindromic repeats (CRISPR) screening platform and use it to identify genetic vulnerabilities in AML cells. We identify 492 AML-specific cell-essential genes, including several established therapeutic targets such as *DOT1L*, *BCL2*, and *MEN1*, and many other genes including clinically actionable candidates. We validate selected genes using genetic and pharmacological inhibition, and chose *KAT2A* as a candidate for downstream study. *KAT2A* inhibition demonstrated anti-AML activity by inducing myeloid differentiation and apoptosis, and suppressed the growth of primary human AMLs of diverse genotypes while sparing normal hemopoietic stem-progenitor cells. Our results propose that KAT2A inhibition should be investigated as a therapeutic strategy in AML and provide a large number of genetic vulnerabilities of this leukemia that can be pursued in downstream studies.

## Highlights

• Optimized CRISPR platform for identification of genome-wide genetic vulnerabilities
• Catalog of genetic vulnerabilities in acute myeloid leukemia cell lines
• KAT2A inhibition induces myeloid differentiation and apoptosis
• KAT2A inhibition arrests the growth of primary AML cells, but not of normal progenitors

## Graphical Abstract

**Figure:**
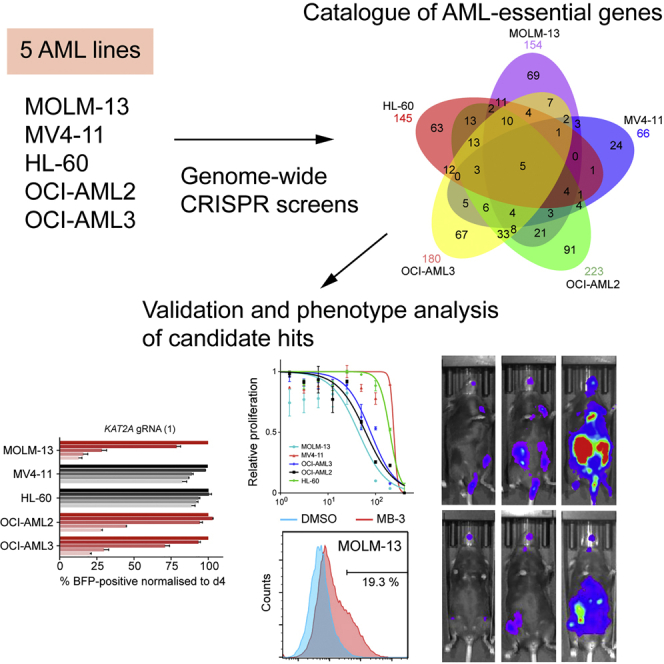

## Teaser

Tzelepis et al. optimize a CRISPR-Cas9-based platform for the performance of genome-wide recessive screens and apply it to identify genetic vulnerabilities of human AML cells. They identify several known therapeutic targets including *BRD4*, *DOT1L*, and *MEN1*, and numerous additional candidates. They provide data proposing *KAT2A* as a potential therapeutic target.

## Introduction

The successful adaptation of the *Streptococcus pyogenes*-derived type II clustered regularly interspaced short palindromic repeats (CRISPR)-Cas system for genome editing is transforming the landscape of genetic research in many organisms (Cho et al., 2013, Cong et al., 2013, Jinek et al., 2012, Mali et al., 2013). Furthermore, the system’s high efficiency and flexibility make it ideal for use in genome-wide recessive genetic screens. In fact, recent proof-of-principle studies have demonstrated the potential of this technology to identify cell-essential genes in mammalian cells (Koike-Yusa et al., 2014, Shalem et al., 2014, Shi et al., 2015, Wang et al., 2014). Previously, this was typically conducted using RNA interference (RNAi) in the form of short interfering RNA (siRNA) or short hairpin RNA (shRNA) libraries (Boutros and Ahringer, 2008, Luo et al., 2009, Schlabach et al., 2008, Silva et al., 2008, Zuber et al., 2011). Such screens have made important contributions to biology, but their success has been moderated by the varying efficiencies of siRNAs/shRNAs for the stringent and specific suppression of target genes required for genome-wide studies (Boutros and Ahringer, 2008). CRISPR-Cas9-based functional genomics may be able to overcome such limitations and, therefore, hold great promise in re-shaping cell-essentiality screens. In cancer research, such screens can be applied to identify genetic vulnerabilities of cancer cells that can be used to develop new anti-cancer treatments. Recent reports on CRISPR screens on several cancer cell lines have demonstrated their power (Hart et al., 2015, Wang et al., 2015).

A human malignancy in urgent need of additional therapies is acute myeloid leukemia (AML), a devastating disorder with a long-term survival rate of less than 30% (Ferrara and Schiffer, 2013). Steady progress in deciphering its molecular pathogenesis has been made over the last few decades with a dramatic acceleration in recent years, particularly as a consequence of advances in cancer genomics (Cancer Genome Atlas Research Network., 2013, Welch et al., 2012). Despite such progress, the therapeutic landscape of AML has changed little for 40 years, with cytarabine still representing the last significant advance (Evans et al., 1961). Although the improved molecular understanding of AML permits some optimism that progress may be forthcoming, an alternative approach for the identification of therapeutic targets is the agnostic interrogation of AML genomes for genetic vulnerabilities using the CRISPR-Cas9 technology. Here, we make significant improvements in this technology and apply these to perform such a screen in AML.

## Results

### Optimization of Genome-wide CRISPR-Cas9 Dropout Screens

**Figure 1:**
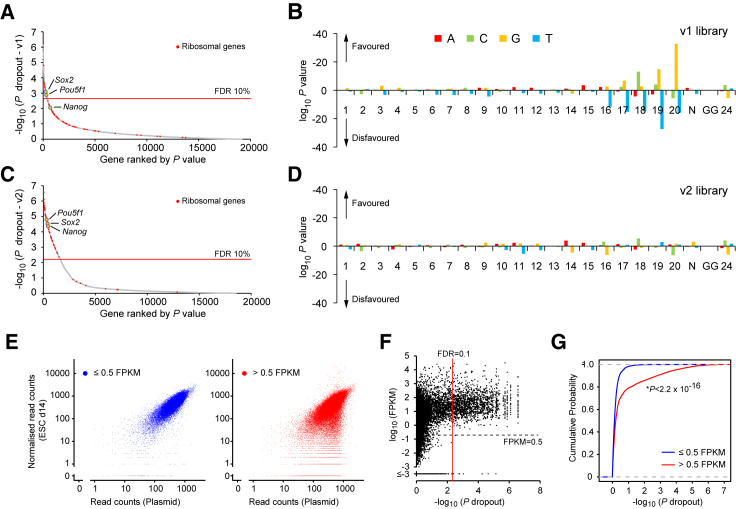
Optimization of CRISPR Dropout Screens and Validation (A–D) Results of dropout screens in mouse ESCs (A and C) and nucleotide-level biases on gRNA efficiency (B and D) identified with version 1 (v1; A and B) and version 2 (v2; C and D) of the mouse genome-wide CRISPR libraries. (E–G) Comparisons between gRNA counts (E) or gene-level significance of dropout and gene expression (F and G). An RNA-seq dataset (GSE44067; Zhang et al., 2013) was used and a cutoff of 0.5 FPKM was applied to distinguish expressed and non-expressed genes. The vast majority of gRNAs targeting non-expressed genes (E, left panel) exhibited equal representation between plasmid and day 14 mouse ESCs, indicating that the library complexity was maintained and that off-target effects were negligible. By contrast, a significant number of expressed genes are under- or over-represented in surviving day 14 ESCs. This is also evident at the gene-level analysis (F and G). The Kolmogorov-Smirnov test was used in (G). See also Figure S1, Table S1, and Data S1.

We and others have demonstrated that the CRISPR-Cas9 system can be adapted for use in functional genetic screens in the form of pooled guide RNA (gRNA) libraries, and that enrichment screens for genes whose inactivation confers resistance to toxins, chemotherapeutics, and targeted cancer treatments can be successfully conducted (Koike-Yusa et al., 2014, Shalem et al., 2014, Wang et al., 2014, Zhou et al., 2014). However, when we applied statistical analyses (Li et al., 2014) to our own genome-wide screen data in mouse embryonic stem cells (ESCs), we were able to identify only a small number of genes depleted to significant levels (Figure 1A). We reasoned that this may be secondary to non-uniform CRISPR-Cas9 efficiency across the large numbers of gRNAs in the library, leading to reduced technical and statistical robustness. To identify factors that affect gRNA efficiency, we first compared nucleotide composition between efficient and inefficient gRNAs in the mouse ESC screen. This analysis revealed strong nucleotide biases between positions 16 and 20 (Figure 1B). These biases also have been observed in human cells (Wang et al., 2014) as well as *Caenorhabditis elegans* (Farboud and Meyer, 2015), suggesting that they may be an intrinsic feature of the current *S. pyogenes* CRISPR-Cas9 platform.

To increase CRISPR-Cas9 efficiency, we first tested a gRNA scaffold optimized for CRISPR imaging (Chen et al., 2013) and found that, consistent with the results shown in a recent report (Dang et al., 2015), gRNAs with the improved scaffold exhibited significantly higher knockout efficiency than those with the conventional scaffold (Figures S1A and S1B). In addition, to generate an optimal gRNA library, we re-designed gRNAs for the mouse genome using a new design pipeline (see Supplemental Experimental Procedures) and generated a murine lentiviral gRNA library (version 2 [v2]) composed of 90,230 gRNAs targeting a total of 18,424 genes (Table S1). We then tested the performance of the v2 library, with regard to depletion (dropout) of genes, with the same experimental setting as with our first version (v1). With the optimized platform, many more genes were depleted at statistically significant levels (360 and 1,680 genes depleted at a false discovery rate [FDR] of 0.1 with the v1 and v2 library, respectively; Figure 1C; Data S1). Furthermore, the nucleotide biases observed in v1 were not observed with the v2 library (Figure 1D), indicating that on-target efficiency prediction (Doench et al., 2016, Wang et al., 2015) may not be necessary with the improved gRNA scaffold. The abundances of gRNAs targeting non-expressed genes (fragments per kilobase of transcript per million mapped reads [FPKM] ≤ 0.5) remained the same as the initial pool (plasmid), whereas large numbers of gRNAs with increased or decreased abundance in surviving ESCs were readily observed for expressed genes (FPKM > 0.5) (Figure 1E). At the gene level, the vast majority of depleted genes were expressed at FPKM > 0.5 in mouse ESCs (Figures 1F and 1G). Taken together, these data show that the sensitivity of our optimized CRISPR dropout screens for detecting cell-essential genes is markedly increased, whereas the off-target effects are negligible.

### Generation and Validation of a Toolkit for CRISPR Dropout Screens in Human Cells

To perform CRISPR dropout screens in cancer cells, we generated a CRISPR functional screening toolkit composed of (1) lentiviral gRNA expression vectors harboring the improved scaffold (Figures S1C–S1E), (2) Cas9 activity reporters (Figures S1F–S1M), and (3) a human genome-wide CRISPR library (v1) consisting of 90,709 gRNAs targeting a total of 18,010 genes (Table S1). We then generated a pool of Cas9-expressing HT-29 colon cancer cells by lentiviral transduction and analyzed Cas9 activity using our reporter system. We found that a proportion of cells did not show detectable Cas9 activity despite growing under antibiotic selection (Figure S2A). Because the presence of Cas9-inactive cells can have an adverse impact on the efficiency of dropout screens, we sub-cloned Cas9-expressing cells and found that this eliminated Cas9-inactive cells (Figure S2B). We consistently observed the presence of Cas9-inactive cells in every cancer cell line tested thus far and found that these cells harbored mutations in the proviral Cas9 coding sequence with an APOBEC3 mutational signature (Hultquist et al., 2011) (Figure S2C). This Cas9-inactive fraction could be reduced by approximately 70% using a lentiviral construct carrying Cas9 upstream, rather than downstream, of the Blasticidin-resistant gene (Figures S1C, S2D, and S2E).

We proceeded to perform dropout screens in clonal Cas9-expressing HT-29 cells. Cells were harvested every 3 days from days 7 to 25 after transduction, and gRNA sequencing was performed (Data S2). As with the mouse ESC screen, a comparison between the screening results and RNA sequencing (RNA-seq) data revealed that the vast majority of depleted genes were expressed in HT-29 cells (Figures S3A and S3B), indicating that off-target effects were also negligible in our human CRISPR library. We identified approximately 2,000 depleted genes at a cutoff of FDR 20% and found that essential biological processes were enriched among them (Figures S3C–S3E).

**Figure 2:**
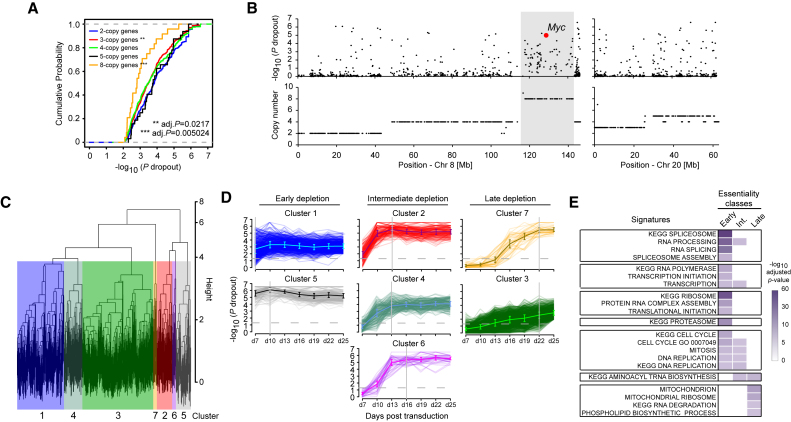
Validation of the Human CRISPR Library in the HT-29 Colon Cancer Cell Line (A) Effects of copy numbers on dropout efficiency in human colon cancer cell line, HT-29. Genes that were significantly depleted at day 25 (FDR < 10%) were grouped according to their copy number. (B) Depletion p values (top) and copy number (bottom) of genes on chromosomes 8 and 20. Note that an eight-copy region containing *Myc* shows a clear distinction in the depletion pattern. Copy number data in HT-29 were obtained from the Catalogue of Somatic Mutations in Cancer (COSMIC) cell line database (http://cancer.sanger.ac.uk/cell_lines/). (C and D) Hierarchical clustering of gene depletion. Genes that were significantly depleted on day 25 (FDR < 10%) were analyzed. (E) Representative gene sets enriched in early intermediate- and late-depletion groups. The full list can be found in Table S2. The Kolmogorov-Smirnov test was used in (A). See also Figures S1–S3, Tables S1, S2, and S3, and Data S2.

Cancer cells often exhibit genomic instability associated with multiple copy number alterations (Beroukhim et al., 2010, Bignell et al., 2010, Zack et al., 2013). To investigate whether copy number affects CRISPR efficiency, we analyzed the distributions of dropout p values for individual genes according to their copy numbers and found no noticeable differences in dropout efficiency for genes with up to five copies (Figure 2A), although genes with three copies showed a modest but statistically significant reduction (adjusted p = 0.0217). By contrast, genes with eight copies, located on the *Myc*-centered distal region on chromosome 8 displayed a depletion pattern, which was very distinct to that of the surrounding region (Figures 2B and S3F). A similar depletion pattern in a continuous chromosome segment was previously observed in a highly amplified region in K562 cells (Wang et al., 2015). These results indicate that most genomic locations are amenable to dropout even when amplified and that knowledge of genome-wide copy number can help interpretation of genome-wide screens.

To investigate the timing of cell-essential gene depletion, we performed a longitudinal dropout analysis using the HT-29 dataset. A quarter of genes that were depleted at day 25 were already depleted by day 7, but the remaining cell-essential genes were depleted during the next 18 days (Figure S3D). An unsupervised cluster analysis of the depletion patterns identified seven clusters (Figure 2C). We further classified these clusters into three groups according to the time point at which depletions reached maximum significance (Figure 2D). Early genes, represented by clusters 1 and 5, were those that reached the highest significance before day 10. The intermediate group (clusters 2, 4, and 6) reached the highest depletion significance on day 13 or 16, whereas the late-depleting group (clusters 3 and 7) showed slow, gradual depletion, which reached maximal significance at later time points. Gene set enrichment analysis (GSEA) revealed dynamic changes in gene signatures over time (Figure 2E; Table S2). Essential biological processes for cell survival were significantly enriched in the early-depletion group, whereas processes involved in proliferation were depleted at early-to-intermediate time points. Genes in the late-depleting group seemed to represent genes whose loss was likely to have a lesser impact on proliferation. For example, this group included genes involved in glycosylphosphatidylinositol anchor biosynthesis, disruption of which leaves cells viable but slower to proliferate (Koike-Yusa et al., 2014). Taken together, CRISPR-Cas9-based dropout screens with our improved lentiviral libraries identified essential genes with high precision and performance in human cancer cells. Our findings establish a technical framework for the performance and interpretation of genome-wide dropout screens using the CRISPR-Cas9 technology.

### Identification of Genetic Vulnerabilities in AML

**Figure 3:**
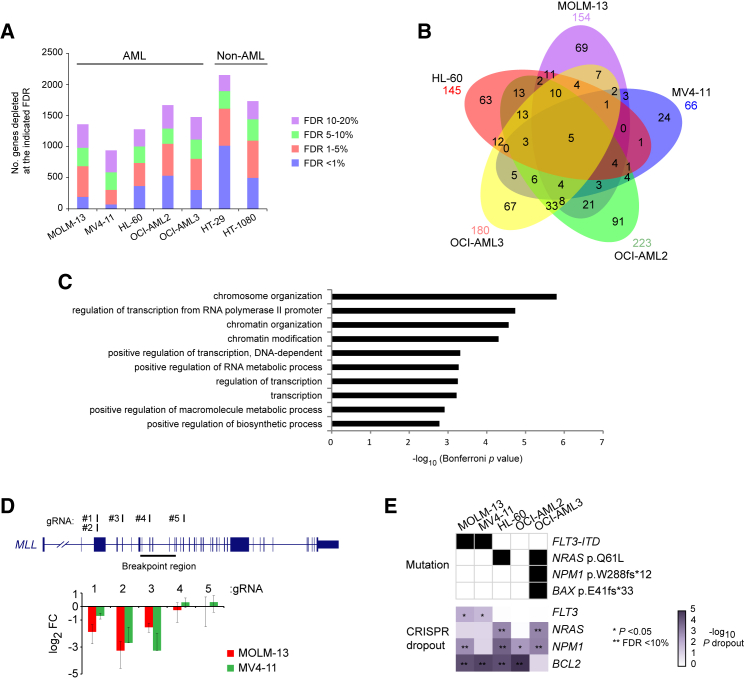
Identification of AML-Cell-Line-Specific Essential Genes (A) Numbers of depleted genes in each of the seven cancer cell lines screened according to FDRs. (B) Venn diagram depicting AML-cell-line-specific cell-essential genes defined as those depleted in at least one AML cell line and not in HT-1080 or HT-29. (C) Gene ontology analysis of the 66 genes essential to three or more AML cell lines. (D) Depletion of five gRNA against *MLL* according to their location relative to the MLL breakpoint region. (E) Depletion of the *FLT3*, *NRAS*, and *NPM1* genes affected by known oncogenic mutations in the specified AML cell lines and of *BCL2*, which was depleted in all AML cell lines except OCI-AML3, which carries a frameshift mutation in *BAX*. See also Figures S4 and S5, Table S3, and Data S2.

Having optimized our platform, we proceeded to perform genome-wide dropout screens in five AML cell lines (MOLM-13, MV4-11, HL-60, OCI-AML2, and OCI-AML3) and the fibrosarcoma line HT-1080 as a second non-AML reference. Similar to HT-29, bulk Cas9-expressing cells included a fraction of cells without Cas9 activity, but single-cell cloning effectively eliminated this population and showed uniform Cas9 activity (Figures S4A and S4B). The karyotypes of the selected Cas9-expressing clones were analyzed for all AML lines and found to agree closely with the published karyotypes of the parental lines (Figures S4C and S4D). The selected clones were transduced with the human CRISPR library, cultured for 30 days, and harvested to determine their gRNA content (Table S3; Data S2). The genome-wide screens, performed using two biological replicates per line, identified circa 1,000–1,500 depleted genes in each AML cell line (Figure 3A). We first determined that significantly depleted genes were almost exclusively derived from those expressed at FPKM > 0.5 in the corresponding cell line (Figures S5A–S5F), showing that off-target effects were very limited and that gene dropouts were likely to have phenotypic consequences on cellular growth and/or survival. We also compared dropout efficiency of known cell-essential genes according to the number of copies of the chromosomes on which they are located and we found no significant difference (Figures S5G–S5K), indicating that Cas9 disrupted genes equally effectively irrespective of copy number in our AML cell lines.

To identify AML-specific vulnerabilities, we focused on genes depleted in one or more AML, but not in either of the non-AML cell lines (Table S3). This analysis identified 66–223 essential genes for each cell line (492 genes in total; Figure 3B), including 66 genes essential to three or more and 5 genes essential to all five AML cell lines. Gene ontology analysis of these genes showed particular enrichment in processes pertaining to chromatin modification and organization and transcriptional regulation (Figure 3C), in keeping with the fact that AML is driven by corrupted epigenetic and transcriptional networks.

We also specifically checked for depletion of driver mutations present in the AML cell lines screened. First, we looked at *MLL* (also known as *KMT2A*) and found that gRNAs targeting the exons upstream of the *MLL* breakpoint region, and therefore predicted to disrupt the *MLL-AF9* and *MLL-AF4* oncogenes, were depleted in both MOLM-13 and MV4-11 (Figure 3D). In addition, gRNAs against *FLT3* and *NRAS* showed specific depletion in cell lines carrying activating mutations in these genes, whereas *NPM1* was depleted in four of the five AML lines including OCI-AML3 (Figure 3E). Interestingly, *BCL2* was depleted in all AML cell lines except OCI-AML3, which carries a *BAX* pE41fs^∗^33 mutation (Figure 3E), suggesting *BAX* mutations as candidate mediators of resistance to BCL2 inhibitors, a promising therapeutic strategy in AML (Chan et al., 2015, Pan et al., 2014).

### Genetic and Pharmacological Validation of the Screening Results

**Figure 4:**
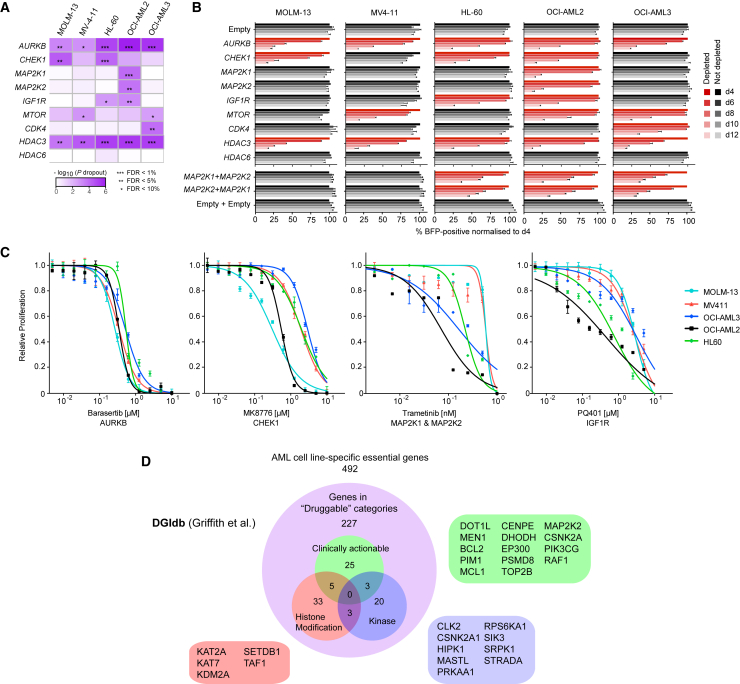
Genetic and Pharmacological Validation of Screen Hits (A) Significance levels for cell essentiality of selected genes in AML cell lines from our dropout screens. (B) Validation of the findings of the screen using a 12-day competitive co-culture assay. Cells were transduced with lentivirus expressing one of two gRNAs per gene, and the BFP-positive fraction was compared with the non-transduced population. Results were normalized to day 4 for each gRNA. Data are shown as mean ± SD (n = 2). The full dataset can be found in Figure S5N. (C) Effects of selected clinical inhibitors on cell growth. The results were normalized to DMSO-treated cells from each cell line cultured in parallel. Data are shown as mean ± SD (n = 3). (D) Drug Gene Interaction database (DGIdb) (Griffith et al., 2013) categorization of AML-specific cell-essential genes into “druggable” categories defined by the DGIdb. Three categories are depicted. Full categorization can be found in Table S4. In the druggable set, representative genes in each of the three categories are listed. See also Figure S5 and Tables S4 and S6.

To validate the results of our screen, we first demonstrated genetically the cell-essential nature of the five dropout genes shared by all AML cell lines (Figure S5M). We then selected eight dropout genes and a control non-dropout gene (*HDAC6*) for targeted inhibition using genetic and pharmacological approaches. We first followed a gene-by-gene knockout approach using the CRISPR-Cas9 system. Two gRNAs (one from our library and one new) were designed per gene, and the relative growth of gRNA-transduced and non-transduced cells were compared in competitive co-culture assays. Results were in close agreement with the findings of our dropout screens (Figures 4A, 4B, and S5N). We then tested the ability of existing clinical compounds to inhibit the growth of the five AML cell lines and again found these to be in consonance with the findings of our genome-wide screens (Figure 4C). *MAP2K1* (also known as *MEK1*) and *MAP2K2* (also known as *MEK2*) are thought to have redundant functions, but OCI-AML2 was sensitive to depletion of either gene. To test *MEK1/2* dependency in the other AML cell lines, we devised a lentiviral dual gRNA expression vector (Figures S1C and S1E) and found that HL-60 and OCI-AML3 were sensitive only to double *MEK1/2* knockout. This differential sensitivity to *MEK1/2* genetic perturbation was mirrored in responses to the dual MEK1/2 inhibitor trametinib.

Reassured by the concordance between the results of our screening and validation experiments, we searched the “druggability” of the 492 genes specifically depleted in our AML cell lines using the Drug Gene Interaction database (DGIdb) (Griffith et al., 2013) and found that 227 (46%) of the genes are in druggable categories (Figure 4D; Table S4). Among these were 33 genes, for which “clinically actionable” compounds are available, which overlap only partially with the “Kinase” and “histone modification” categories. However, the majority of genes in the druggable categories were not previously considered potential therapeutic targets (Figure 4D). Of note, at least 12 dropout genes, including BRD4, that were essential to at least three AML cell lines, as well as to HT-29 and HT-1080, are targets of clinical inhibitors (Table S4), indicating that “pan-essential” genes should not be dismissed as potential therapeutic targets.

### Selection of Rational Therapeutic Development Targets

**Figure 5:**
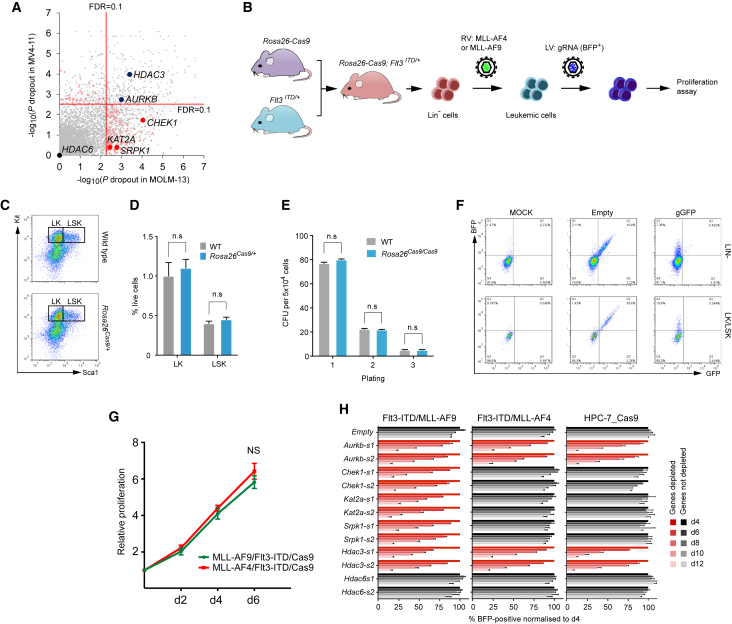
Differential Vulnerabilities between MLL-AF4- and MLL-AF9-Driven Leukemias (A) Comparison of dropout p values between MOLM-13 and MV4-11. *AURKB* and *HDAC3* were significantly depleted in both lines, but *HDAC6* was not in either line. In contrast, *CHEK1*, *KAT2A*, and *SRPK1* were depleted only in MOLM-13. Genes that are specifically depleted in either cell line (FDR < 0.1) but not in either non-AML cell line are highlighted in pale red. (B) Schematic of CRISPR-based validation of genotype-specific essentialities using ex vivo mouse leukemia model. (C and D) Normal percentages of LK (Lin^−^/Kit^+^) and LSK (Lin^−^/Sca1^+^/Kit^+^) hemopoietic stem-progenitor cells were identified in the bone marrow of *Rosa26*^*Cas9/+*^ mice. Data are shown as mean ± SD (n = 3). (E) Colony-forming assays of bone marrow cells derived from WT and *Rosa26*^*Cas9/Cas9*^ mice, showing no differences in replating ability of *Rosa26*^*Cas9/Cas9*^ cells compared with WT. (F) Validation of Cas9 activity in Lin^−^ or LK/LSK cells from *Rosa26*^*Cas9/+*^ mice using the Cas9 activity reporter. (G) Growth kinetics of primary Lin^−^ cells from *Flt3*^*ITD/+*^*;Rosa26*^*Cas9/+*^ mice transformed with a retrovirus expressing MLL-AF4 or MLL-AF9. Data are shown as mean ± SD (n = 4). (H) Competitive co-culture assay showing oncogene-specific vulnerabilities in the ex vivo leukemia model. As a normal cell control, non-leukemic HPC-7 mouse hematopoietic cells were used. Results were normalized to day 4 for each gRNA. Data are shown as mean ± SD (n = 3). The Student’s t test was performed in (D) and (E). Two-way ANOVA was performed in (G). See also Figure S6 and Table S6.

Our approach thus far has enabled us to define a set of genes that are essential to AML, but not to either of two solid cancer cell lines. However, it is probable that some of these AML-essential genes are also essential to normal blood cells including hemopoietic stem cells (HSCs), and such genes may not represent plausible therapeutic targets. Because currently no methods are available for systematic identification of essential genes in normal HSCs, we took an alternative strategy to identify therapeutic targets. In particular, we hypothesized that genes displaying cell line or oncogene specificity were less likely to cause toxicity to normal HSCs but could still be relevant to multiple AML genotypes. To do this, we compared the cell-essential genes of the MOLM-13 and MV4-11 cell lines. These both carry an internal tandem duplication in the *FLT3* gene (*FLT3-ITD*) and multiple copies of chromosome 8, and exhibit comparable response to DOT1L and BRD4 inhibitors, but harbor the distinct, but related, fusion genes, *MLL-AF9* (MOLM-13) and *MLL-AF4* (MV4-11), known to directly establish leukemogenic transcriptional programs. Looking at the depleted genes (FDR < 0.2), we noted that MOLM-13 and MV4-11 showed significant overlap, but also many differences (Figure 5A; Table S3). Among these differentially essential genes, we selected two druggable genes for further study: the histone acetyltransferase gene *KAT2A* (also known as *GCN5*) and the spliceosome kinase gene *SRPK1*. We also chose *CHEK1*, a known therapeutic target (Daud et al., 2015, Zabludoff et al., 2008), as a control gene with a similar depletion pattern in both. In addition, we chose *AURKB* and *HDAC3* as control essential genes to both and *HDAC6* as essential to neither cell line (Figure 5A).

To test whether the observed essentialities for *KAT2A* and *SRPK1* are indeed attributable to the different MLL oncogenic fusions rather than other differences between MOLM-13 and MV4-11, we developed a genetically defined experimental model (Figure 5B). First, we generated mice expressing Cas9 constitutively under the control of the ubiquitous *EF1a* promoter from the *Rosa26* locus (Figure S6). *Rosa26*^*Cas9/+*^ mice reproduced in the expected Mendelian ratios exhibited normal long-term survival and had normal hemopoietic stem and progenitor cell numbers, and normal proportions of blood cell subtypes (Figures 5C, 5D, S6G, and S6H). Cas9-expressing stem-progenitor cells exhibited comparable colony-forming and serial replating activity to wild-type (WT) cells and displayed highly efficient Cas9 function (Figures 5E and 5F). These results indicate that Cas9 expression has no detectable effect on the hematopoietic system and that any phenotype observed in gRNA-expressing cells is likely caused by genetic perturbation of a target gene. *Rosa26*^*Cas9/+*^ mice were crossed to *Flt3*^*ITD/+*^ mice (Lee et al., 2007), and lineage-negative hemopoietic progenitors from *Rosa*^*Cas9/+*^*;Flt3*^*ITD/+*^ double transgenic mice were transduced with retroviral vectors expressing *MLL-AF4* (Montes et al., 2011) or *MLL-AF9* (Dawson et al., 2011) and cultured in vitro for 10–12 days, displaying similar exponential growth rates and a myeloid phenotype (Figure 5G). The cells were then independently transduced with individual lentiviruses carrying one of two gRNAs against the *Kat2a*, *Srpk1*, *Aurkb*, *Chek1*, *Hdac3*, and *Hdac6* genes. In keeping with the results of our screen, this revealed significant differences in cell growth between MLL-AF4- and MLL-AF9-driven cells transduced with *Chek1*, *Kat2a*, and *Srpk1* gRNAs, whereas *Aurkb* and *Hdac3* gRNAs were equally effective against both cell types (Figure 5H). In addition, we tested the essentiality of each gene to the non-leukemic mouse multipotent *HPC-7* cells, which represent an early blood stem-progenitor cell and are capable of generating functional hematopoietic cells in vivo (Wilson et al., 2016). As shown in Figure 5H, all three of the MLL-AF9-specific essential genes had no effects on proliferation of HPC-7 cells. Taken together, these results support our strategy to use oncogene-specific essentialities for candidate prioritization and provide genetic evidence that *KAT2A* and *SRPK1* are attractive drug targets. We chose to investigate *KAT2A* further because this gene is essential to three of the five AML cell lines studied (MOLM-13, OCI-AML2, and OCI-AML3), and as such may be relevant to a wider group of AML patients. Of note, the OCI-AML3 line carries a mutation in *NPM1*, which is also found in 25%–35% of primary AMLs.

### Mechanistic Insights into the Effects of KAT2A Inhibition in AML

**Figure 6:**
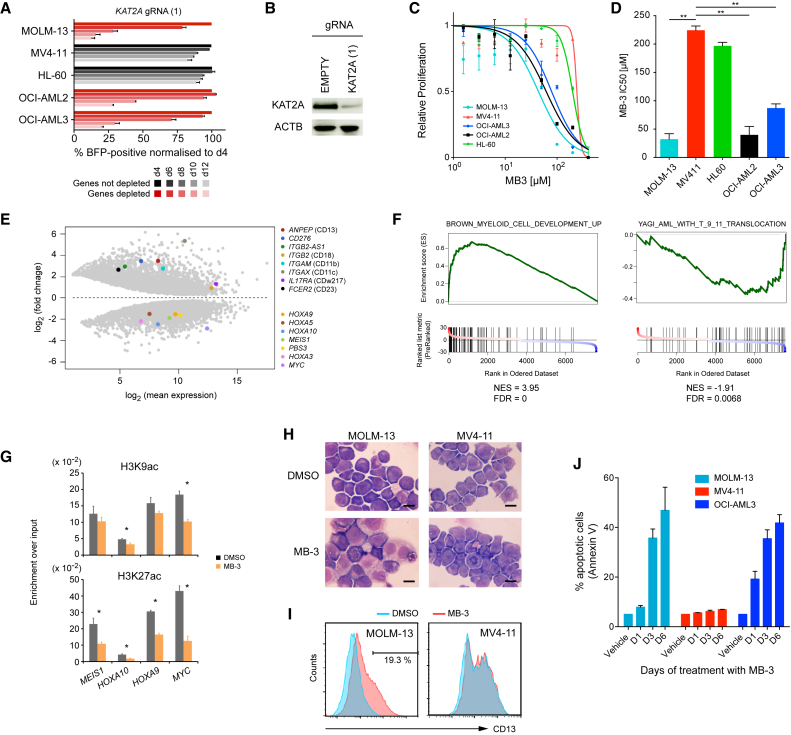
KAT2A Suppression Induces Myeloid Differentiation and Apoptosis (A) CRISPR-based validation of KAT2A depletion in the five AML cell lines. Full results can be found in Figure S5N. (B) Western blot analysis of KAT2A expression in MOLM-13 targeted by *KAT2A*-specific gRNA. (C and D) Drug response (C) and 50% inhibitory concentration (IC_50_) values (D) of the five AML cell lines treated with the KAT2A inhibitor MB-3. (E) Differentially expressed genes in MB-3-treated MOLM-13. AML program genes (downregulated) and myeloid marker genes (upregulated) are highlighted. (F) Gene set enrichment analysis (GSEA) showing significant enrichment for the AML program and myeloid differentiation. (G) Histone H3 acetylation status of genes downregulated by MB-3 treatment using ChIP-qPCR assay. (H and I) Microscopic (H) and flow cytrometric (I) analyses of myeloid differentiation after 24-hr treatment with 100 μM MB-3. No changes were observed in MB-3-insensitive MV4-11 cells. Scale bar, 10 μm. (J) Increased apoptosis after treatment with 100 μM MB-3. Data are shown as mean ± SD (n = 3 in C, D, and J; n = 2 in G). The Student’s t test was performed in (D) and (G). ^∗^p < 0.05; ^∗∗^p < 0.01. See also Figure S5 and Table S6.

First, using two separate gRNAs, we confirmed that genetic disruption of *KAT2A* reduced the growth of MOLM-13, OCI-AML2, and OCI-AML3, but not MV4-11 and HL-60 (Figures 6A and S5N). We confirmed that targeting with *KAT2A*-specific gRNA was associated with significantly reduced levels of KAT2A protein (Figure 6B). We then tested the effects of the KAT2A inhibitor MB-3 (Biel et al., 2004) on the growth of these lines and found that drug response mirrored the genetic validation studies (Figures 6C and 6D). To obtain mechanistic insights into the molecular effects of pharmacological KAT2A inhibition, we performed RNA-seq analysis on the sensitive MOLM-13 line after a 48-hr exposure to MB-3. We identified significant changes in gene expression including downregulation of genes associated with the MLL-AF9 leukemogenic program such as *HOXA9*, *HOXA10*, *MEIS1*, and *MYC*, and concomitant upregulation of genes associated with myeloid differentiation including *ANPEP* (CD13), *ITGB2* (CD18), *ITGAM* (CD11b), and *IL17RA* (CD23) (Figures 6E and 6F). In keeping with these effects being the results of reduced KAT2A function, we confirmed that using chromatin immunoprecipitation (ChIP)-qPCR MB-3 led to reduction of the acetylation level at lysine-9 and lysine-27 of histone H3 at the downregulated gene loci: *HOXA9*, *HOXA10*, *MEIS1*, and *MYC* (Figure 6G). Microscopic and flow cytometry analyses of MOLM-13 cells after a 48 hr exposure to MB-3 confirmed monocytic-macrophage differentiation (Figure 6H) and increased CD13 surface expression (Figure 6I), whereas neither was observed in the MB-3-insensitive MV4-11 cells. Furthermore, prolonged incubation with MB-3 caused a marked increase in apoptosis of MOLM-13 and OCI-AML3, but not MV4-11 (Figure 6J). Taken together, these results indicated that KAT2A inhibition suppresses AML cell proliferation through inhibition of leukemogenic transcriptional programs and induction of differentiation leading to cell death by apoptosis.

### Clinical Potential of KAT2A Inhibition in AML Therapy

**Figure 7:**
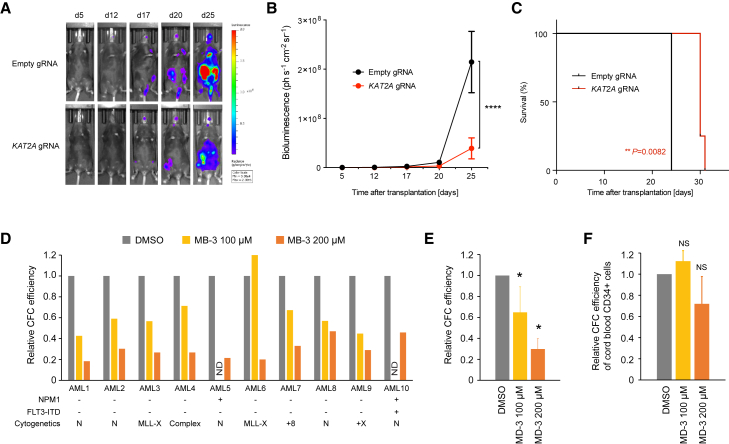
KAT2A Inhibition Shows Suppression of Leukemic Cell Growth In Vivo and Human Primary AML Cells (A) Bioluminescence imaging of mice transplanted luciferase-labeled gRNA-transduced MOLM-13 cells at indicated time points. (B) Quantification of luminescence. ^∗∗∗∗^p < 0.0001. (C) Kaplan-Meier plot showing survival of mice transplanted with MOLM-13 expressing the indicated gRNA. Log rank test was performed. (D and E) Colony-forming cell (CFC) assay of 10 primary AMLs of diverse genotypes with 100 and 200 μM MB-3. Detailed information can be found in Table S5. Mean values of 10 samples are shown in (E). Error bars represent SD. ^∗^p < 0.05. (F) CFC efficiency of CD34^+^ human cord blood cells (n = 4). The Student’s t test was performed in (B), (E), and (F). See also Table S5. N, normal karyotype; ND, not determined.

We next investigated whether KAT2A inhibition reduces cell proliferation in vivo. We first introduced the luciferase gene into MOLM-13-Cas9 cells and then transduced the cells with either an empty gRNA scaffold or a gRNA targeting the *KAT2A* gene. After 3 days of puromycin selection, transduced cells were transplanted into immunocompromised *Rag2*^*−/−*^*;Il2rg*^*−/−*^ mice, which were then imaged for bioluminescence until death. We found that *KAT2A* disruption was associated with a significant reduction in AML cell expansion (Figures 7A and 7B) and prolongation of mouse survival (Figure 7C), indicating that KAT2A inhibition suppresses AML cell proliferation in vivo. Encouraged by these results, we proceeded to test the effects of MB-3 on primary human AML cells. Treatment of 10 primary AMLs of diverse genotypes (Table S5) with MB-3 led to significant reduction of colony formation in methylcellulose media at both 100 and 200 μM concentration (Figures 7D and 7E). By contrast, the colony-forming cell (CFC) efficiency of CD34^+^ human cord blood cells was not significantly affected by 100 or 200 μM MB-3 (Figure 7F). Taken together, our results show that KAT2A inhibition does not exhibit adverse effects on hematopoietic stem-progenitor cells and offers itself as a potential anti-AML therapeutic strategy for future studies.

## Discussion

Despite important advances in understanding their genomic and molecular pathogenesis, many cancers including AML continue to represent unmet clinical challenges (Cancer Genome Atlas Research Network., 2013, Döhner et al., 2015). It is therefore crucial to develop additional therapeutic strategies by identifying vulnerabilities in cancer cells. This can be achieved by either hypothesis-driven mechanistic studies or hypothesis-free unbiased genetic screening. In AML, recent detailed mechanistic studies have identified *DOT1L* as a vulnerability of MLL-rearranged leukemia (Bernt et al., 2011), and both a mechanistic and an RNAi-based epigenetics-focused screen identified *BRD4* as a therapeutic target against AMLs of different genotypes (Dawson et al., 2011, Zuber et al., 2011). Drug development against these targets has rapidly progressed and their therapeutic efficacy is now being tested in clinical trials. Nevertheless, despite these successes, AML remains a lethal disease for most patients, and a complete set of genetic vulnerabilities for this and other cancers remains unknown, leaving many candidates with a therapeutic potential undiscovered.

To this end, we optimized and validated a robust CRISPR-Cas9 platform for the performance of genome-wide essentiality screens and applied this to catalog genetic vulnerabilities in AML. Our results have not only confirmed known therapeutic targets but also revealed a large number of genetic vulnerabilities in the AML cell lines studied, many of which represent plausible direct or indirect targets for drug development. Importantly, the unbiased nature of genome-wide screens such as ours makes them a powerful instrument for the identification of such targets, which is both orthogonal and complementary to mechanistic studies of disease pathogenesis and also able to reveal both intuitive and non-intuitive vulnerabilities.

Nevertheless, not all genetic vulnerabilities represent viable therapeutic targets. An important hurdle in selecting these is the real possibility that any genes essential to AML cells may also be essential to normal hemopoietic and/or non-hemopoietic cells, making their pharmacological inhibition harmful. To select targets that are likely to exhibit minimal adverse effects and thus have a higher likelihood of success in drug development, we applied a differential essentiality filter to our screen dataset and identified and characterized a potential AML therapeutic target, namely *KAT2A*. Genetic or pharmacological suppression of KAT2A did not show detectable adverse effects in either mouse HPC-7 hematopoietic precursor cell line or human cord blood CD34^+^ cells, further supporting that our approach was valid. It would be important to identify any toxic effects on hemopoietic stem-progenitor cells using a potent and bioavailable KAT2A inhibitor. Of course, in the absence of comprehensive datasets from other normal cell types, we cannot rule out the possibility that KAT2A suppression can cause side effects, and the generation of such datasets would significantly enhance our ability to predict clinical toxicity and identify the most promising therapies. It is, however, noteworthy that at least a dozen targets that have been in clinical use already were essential to cell types other than AML, suggesting that valuable targets can be found even among genes within this category and may potentially have a broad spectrum of antitumor activity.

Notwithstanding limitations in predicting clinical toxicity, our results demonstrate that *KAT2A* inhibition induces cellular differentiation and apoptosis of AML cells. Although the precise molecular basis of these effects will need to be investigated in future studies, the transcriptional changes associated with *KAT2A* inhibition suggest that the effects may be secondary to inhibition of leukemogenic transcriptional programs, in a manner reminiscent of BRD4 and DOT1L inhibition (Bernt et al., 2011, Dawson et al., 2011). *KAT2A* encodes a histone lysine acetyltransferase that functions within the multi-protein transcriptional co-activator complexes SAGA (Spt-Ada-Gcn5-acetyltransferase) or ATAC (Ada2a-containing); the former predominantly localizes at a subset of active promoters, whereas the latter localizes at distinct active promoters and enhancers (Krebs et al., 2011). As such, KAT2A influences diverse transcriptional programs and participates in multiple developmental and cellular processes (Wang and Dent, 2014). It has been shown that leukemia induction by MLL-AF9 requires the Myb-p300 interaction, which is thought to be responsible for the methylation-to-acetylation switch at the lysine-27 residue of histone H3 upon MLL-AF9 expression in HSCs (Pasini et al., 2010). One hypothesis is that a KAT2A-containing complex serves as a transcriptional coactivator that is also recruited to the target sites by MLL-AF9 and activates and/or maintains the leukemic transcriptional program. Alternatively, KAT2A might maintain the leukemic program through acetylation of non-histone proteins as exemplified by direct acetylation of the RUNX1/MDS1/EVI1 (Senyuk et al., 2003) and E2A-PBX1 (Holmlund et al., 2013) fusion oncoproteins by KAT2A and its homolog KAT2B (also known as PCAF). Further work is required to investigate the molecular function of KAT2A and determine the full therapeutic potential of this finding.

Our work demonstrates the power of unbiased genome-wide screens to catalog a comprehensive set of genetic vulnerabilities in cancer cells. Such catalogs enable not only the rapid identification of new targets and development of therapeutic strategies, but also generate hypotheses pertinent to the study of molecular mechanisms underlying tumorigenesis.

## Experimental Procedures

All reagents and detailed methods are described in the Supplemental Information.

### Plasmids, Cell Lines, Mouse Lines, and Reagents

Guide RNA expression vectors with the improved scaffold, pKLV2-U6gRNA5(BbsI)-PKGpuro2ABFP-W and pKLV2.2-h7SKgRNA5(SapI)-U6gRNA5(BbsI)-PGKpruo2ABFP-W, for a single and dual gRNA expression, respectively, were generated in this study and have been deposited with Addgene. The optimized human and murine CRISPR libraries were also available through Addgene. Guide RNA sequences used in a gene-by-gene approach are listed in Table S6. All AML cell lines (MOLM-13, MV4-11, HL-60, OCI-AML2, and OCI-AML3), colon cancer cell line HT-29, and fibrosarcoma cell line HT-1080 were obtained from the Sanger Institute Cancer Cell Line Panel and were mycoplasma free. Cas9-expressing cell lines were generated by lentiviral transduction using pKLV2-EF1aBsd2ACas9-W, and Cas9 activity in individual subclones was tested using a lentiviral reporter pKLV2-U6gRNA(gGFP)-PGKBFP2AGFP-W. A Cas9-expressing mouse line was generated by inserting the human *EF1a* promoter-driven Cas9 expression cassette into the *Rosa26* locus in mouse ESC line JM8 (Pettitt et al., 2009) and is kept in the C57BL/6N background. See also Supplemental Information. All animal studies were carried out in accordance with the Animals (Scientific Procedures) Act 1986 (UK) and approved by the Ethics Committee at the Sanger Institute.

### Generation of Genome-wide Mutant Libraries and Screening

A total of 3.0 × 10^7^ cells were transduced with a predetermined volume of the genome-wide gRNA lentiviral supernatant. Two days after transduction, the cells were selected with puromycin for 4 days and further cultured. For HT-29, approximately 1 × 10^8^ cells were harvested every 3 days between day 7 and day 25 post-transduction. The AML cell lines and HT-1080 were harvested on day 25 post-transduction. See also Supplemental Information.

### gRNA Competitive Proliferation Assay

Cas9-expressing cells were transduced with a lentivirus expressing a gene-specific gRNA, and the percentage of blue fluorescent protein (BFP)-positive cells was measured between days 4 and 12 post-transduction and normalized to the percentage of BFP-positive cells at day 4. See also Supplemental Information.

### Drug and Proliferation Assays

A total of 3 × 10^4^ human or primary mouse cells were plated onto 96-well plates with vehicle or the indicated concentrations of compounds. Plates were measured 72 hr post-treatment using CellTiter 96 AQueous Non-Radioactive Cell Proliferation Assay (Promega). See also Supplemental Information.

### Adult Primary Leukemia and Cord Blood Sample Analysis

All human AML and cord blood samples were obtained with informed consent under local ethical approval (REC 07-MRE05-44). Primary AML cells or cord-blood-derived CD34^+^ cells were tested for colony-forming efficiency in H4435 semi-solid medium (Stem Cell Technologies) in the presence of the indicated concentration of MB3 or DMSO. Colonies were counted by microscopy 10–11 days (AML cells) or 12–14 days (CD34^+^ cells) after plating. See also Supplemental Information.

### Statistical Analysis

Statistical analyses performed and the numbers of replicates were mentioned in the associated figure legends. Differences were considered significant for p < 0.05.

## Author Contributions

G.S.V., K.T., and K.Y. conceived the study and designed the experiments. Y.L. and K.Y. designed the mouse and human gRNA libraries. H.K-Y., S.O., and K.Y. generated the CRISPR toolkit and the Cas9 transgenic mice, and performed CRISPR screens in mouse ESCs and HT-29. K.T. performed CRISPR screens in the AML cell lines and conducted the validation and drug sensitivity assays with help from E.D.B., E.M., and K.Y. K.Y. performed the large-scale data analyses with help from K.T., F.I., E.D.B., H.P., and G.S.V. E.M., A.M., M.M., M.G., O.M.D., T.M., M.P., and J.C. performed cell culture and mouse experiments. O.M.D. and B.C. performed CRISPR screens for HT-1080. V.G. and U.M. generated and analyzed Cas9-expressing HT-29. M.L. performed analysis of RNA-seq data. J.S.-R. and B.J.P.H. contributed to study strategy, technical and analytical aspects. C.P. conceived and designed analysis of KAT2A inhibition in AML cell lines and in primary patient and cord blood samples, and performed the experiments with help from K.T. and A.F.D. S.T. supported RNA-seq analysis of MB3-treated MOLM-13 cells. P.G. and B.J.P.H. contributed with AML patient samples. K.T., E.D.B., G.S.V., and K.Y. wrote the paper with input from all authors.

## Conflict of Interest

G.S.V. is a consultant for Kymab and received an educational grant from Celgene.

## Accession Numbers

The accession numbers for the CRISPR data reported in this paper are European Nucleotide Archive (http://www.ebi.ac.uk/ena): ERP006734 (mouse ESCs), ERP005600 (HT-29), and ERP008475 (AML and HT-1080). The accession numbers for the RNA-seq data reported in this paper are European Nucleotide Archive: ERP006662 and ERP003933. The CRISPR toolkit and the CRISPR libraries are available from Addgene.

## Supplemental Information

Document S1. Supplemental Experimental Procedures, Figures S1–S6 and Tables S4–S6

Table S1. Lists of gRNAs in the Mouse v2 and Human v1 CRISPR Libraries, Related to Figures 1 and 2

Table S2. Genes and Enriched Pathways in Each Cluster Identified in the Time-Course HT-29 Dropout Experiment, Related to Figure 2

Table S3. Human CRISPR Dropout Summary, Related to Figures 2 and 3 The statistical result on gene depletion in all human cancer cell lines used in this study is shown. Depletion p values, depletion FDR, and RNA-seq counts (log_10_-transformed FPKM) are shown for each gene in each cell line. Note that genes whose RNA-seq count is ≤0.001 FPKM are all given a value of −3.5 as a log_10_-transformed value. Summaries of depleted genes in each human cancer cell line at FDR 20% or 10% are also shown in separate spreadsheets.

Data S1. Mouse CRISPR Screen Data, Related to Figure 1

Data S2. Human CRISPR Screen Data, Related to Figures 2 and 3

Document S2. Article plus Supplemental Information
