# Supplementary material for: A CRISPR Dropout Screen Identifies Genetic Vulnerabilities and Therapeutic Targets in Acute Myeloid Leukemia
Source: Cell Rep. 2016 Oct 18;17(4):1193–205. doi: 10.1016/j.celrep.2016.09.079 (PMC5081405; doi:10.1016/j.celrep.2016.09.079)
Supplement: Document S1. Supplemental Experimental Procedures, Figures S1–S6 and Tables S4–S6 [file mmc1.pdf]

Cell Reports, Volume 17

Supplemental Information

A CRISPR Dropout Screen Identifies Genetic Vulnerabilities and Therapeutic Targets in Acute Myeloid Leukemia

Konstantinos Tzelepis, Hiroko Koike-Yusa, Etienne De Braekeleer, Yilong Li, Emmanouil Metzakopian, Oliver M. Dovey, Annalisa Mupo, Vera Grinkevich, Meng Li, Milena Mazan, Malgorzata Gozdecka, Shuhei Ohnishi, Jonathan Cooper, Miten Patel, Thomas McKerrell, Bin Chen, Ana Filipa Domingues, Paolo Gallipoli, Sarah Teichmann, Hannes Ponstingl, Ultan McDermott, Julio Saez-Rodriguez, Brian J.P. Huntly, Francesco Iorio, Cristina Pina, George S. Vassiliou, and Kosuke Yusa

Figure S1

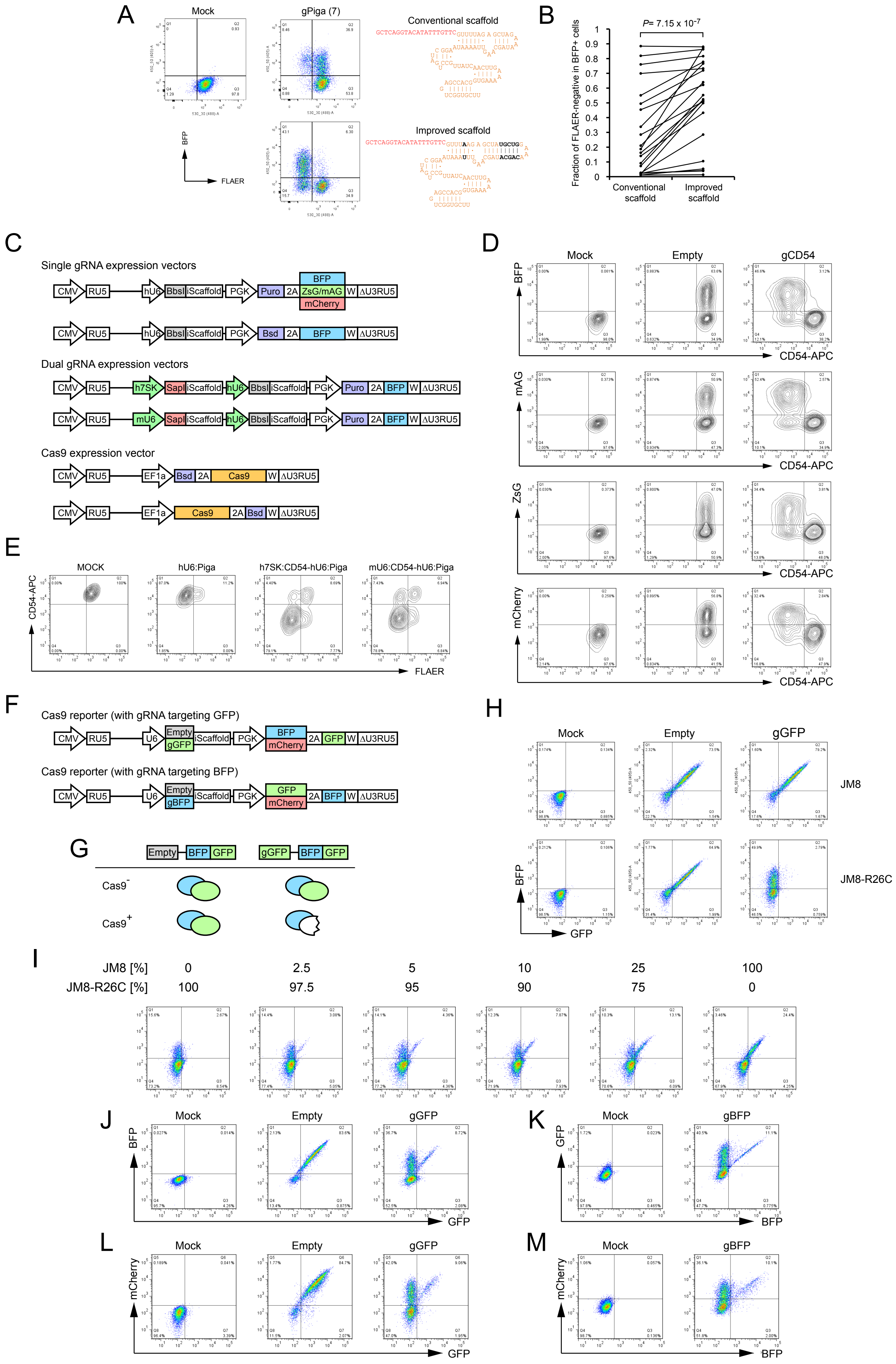
A
Mock
gPiga (7)
BFP
FLAER
Conventional scaffold
Improved scaffold
B
P= 7.15 x 10-7
Fraction of FLAER-negative in BFP+ cells
Conventional scaffold
Improved scaffold
C
Single gRNA expression vectors
CMV RU5 hU6 BbsI iScaffold PGK Puro 2A BFP ZsG/mAG mCherry W ΔU3RU5
CMV RU5 hU6 BbsI iScaffold PGK Bsd 2A BFP W ΔU3RU5
Dual gRNA expression vectors
CMV RU5 h7SK SapI iScaffold hU6 BbsI iScaffold PGK Puro 2A BFP W ΔU3RU5
CMV RU5 mU6 SapI iScaffold hU6 BbsI iScaffold PGK Puro 2A BFP W ΔU3RU5
Cas9 expression vector
CMV RU5 EF1a Bsd 2A Cas9 W ΔU3RU5
CMV RU5 EF1a Cas9 2A Bsd W ΔU3RU5
D
Mock
Empty
gCD54
BFP
mAG
ZsG
mCherry
CD54-APC
E
MOCK
hU6:Piga
h7SK:CD54-hU6:Piga
mU6:CD54-hU6:Piga
CD54-APC
FLAER
F
Cas9 reporter (with gRNA targeting GFP)
CMV RU5 U6 Empty gGFP iScaffold PGK BFP mCherry 2A GFP W ΔU3RU5
Cas9 reporter (with gRNA targeting BFP)
CMV RU5 U6 Empty gBFP iScaffold PGK GFP mCherry 2A BFP W ΔU3RU5
G
Empty BFP GFP
gGFP BFP GFP
Cas9-
Cas9+
H
Mock
Empty
gGFP
JM8
JM8-R26C
BFP
GFP
I
JM8 [%] 0 2.5 5 10 25 100
JM8-R26C [%] 100 97.5 95 90 75 0
J
Mock
Empty
gGFP
BFP
GFP
K
Mock
gBFP
GFP
BFP
L
Mock
Empty
gGFP
mCherry
GFP
M
Mock
gBFP
mCherry
BFP

Figure S1 (Related to Figures 1 and 2). **CRISPR functional screening toolkit. A**,**B**, Comparison of gRNA scaffolds on knockout efficiency. Twenty three gRNAs targeting the *Piga* gene were individually expressed with the conventional (top-right in **A**) or the optimised (bottom-right in **A**) scaffold in Cas9-expressing mouse ESCs and GPI-anchored protein expression was analysed by flow cytometry, following FLAER staining. FLAER is FITC-labelled mutant aerolysin, which stains cells expressing GPI-anchored proteins. The improved scaffold exhibited significantly higher gene knockout efficiency (**B**). Wilcoxon's signed-rank test was performed. **C-E**, **Screening kit for mutagenesis. C**, Schematic of lentiviral single or dual gRNA expression vectors with the improved scaffold (iScaffold) and different fluorescent proteins, and lentiviral Cas9 expression vectors. CMV, CMV promoter; RU5, 5' long terminal repeat; hU6, human U6 promoter; h7SK, human 7SK promoter; mU6, mouse U6 promoter; BbsI and SapI, guide RNA cloning site with BbsI and SapI, respectively; PGK, mouse *Pgk1* promoter; puro, puromycin resistant gene; Bsd, Blasticidin resistant gene; 2A, Thosea asigna virus 2A peptides; BFP, blue fluorescent protein; ZsG, *Zoanthus* sp. green fluorescent protein; mAG, monomeric Azami-Green fluorescent protein; mCherry, monomeric red fluorescent protein; W, Woodchuck Hepatitis Virus posttranscriptional regulatory element; ΔU3RU5, self-inactivating 3' LTR; EF1a, intron-containing human elongation factor 1a promoter; Cas9, codon-optimised *Streptococcus pyogenes* Cas9, double-NLS-tagged (Cong et al., 2013). **D**, Flow cytometry analysis of ESCs transduced with a lentivirus carrying gRNA targeting *CD54*. ESCs were stained with APC-conjugated anti-CD54 6 days post transduction. All lentiviral gRNA expression vectors produced equal knockout phenotype in a corresponding colour channel. **E**, Flow cytometry analysis of ESCs transduced with a lentivirus carrying two gRNAs targeting *CD54* and *Piga*. **F-M**, **Screening kit for Cas9 functional assay. F**, Schematic of the lentiviral vectors for Cas9 functional assay. gGFP, guide RNA targeting GFP coding sequence; gBFP, guide RNA targeting BFP coding sequence; Empty, the original BbsI cloning site. **G**, Schematic showing expected fluorescent protein expression patterns in Cas9+ and Cas9- cells. When the empty vector is used, both GFP and BFP will be detected regardless of Cas9 function. When the vector carrying the guide RNA targeting GFP is used, only BFP is detected when Cas9 is active, whereas both fluorescent proteins can be detected in Cas9-inactive cells. **H,** The expected fluorescent expression patterns were confirmed in wild-type and Cas9-expressing mouse ESCs by flow cytometry analysis 3 days after transduction. **I**, Detection of Cas9-inactive cells using the reporter system. Wild-type and *Rosa26*$^{Cas9/+}$ ESCs were mixed at the indicated ratio and transduced with the reporter virus. Flow cytometry analysis was performed 3 days after transduction. A contamination of 2.5% wild-type cells was clearly detected. **J-M**, Example flow cytometry profiles of Cas9 functional assay with different colour combinations. A guide RNA targeting GFP (**J,L**) or BFP (**K,M**) were used. The bulk Cas9-expressing HT-29 cells were used.

Figure S2

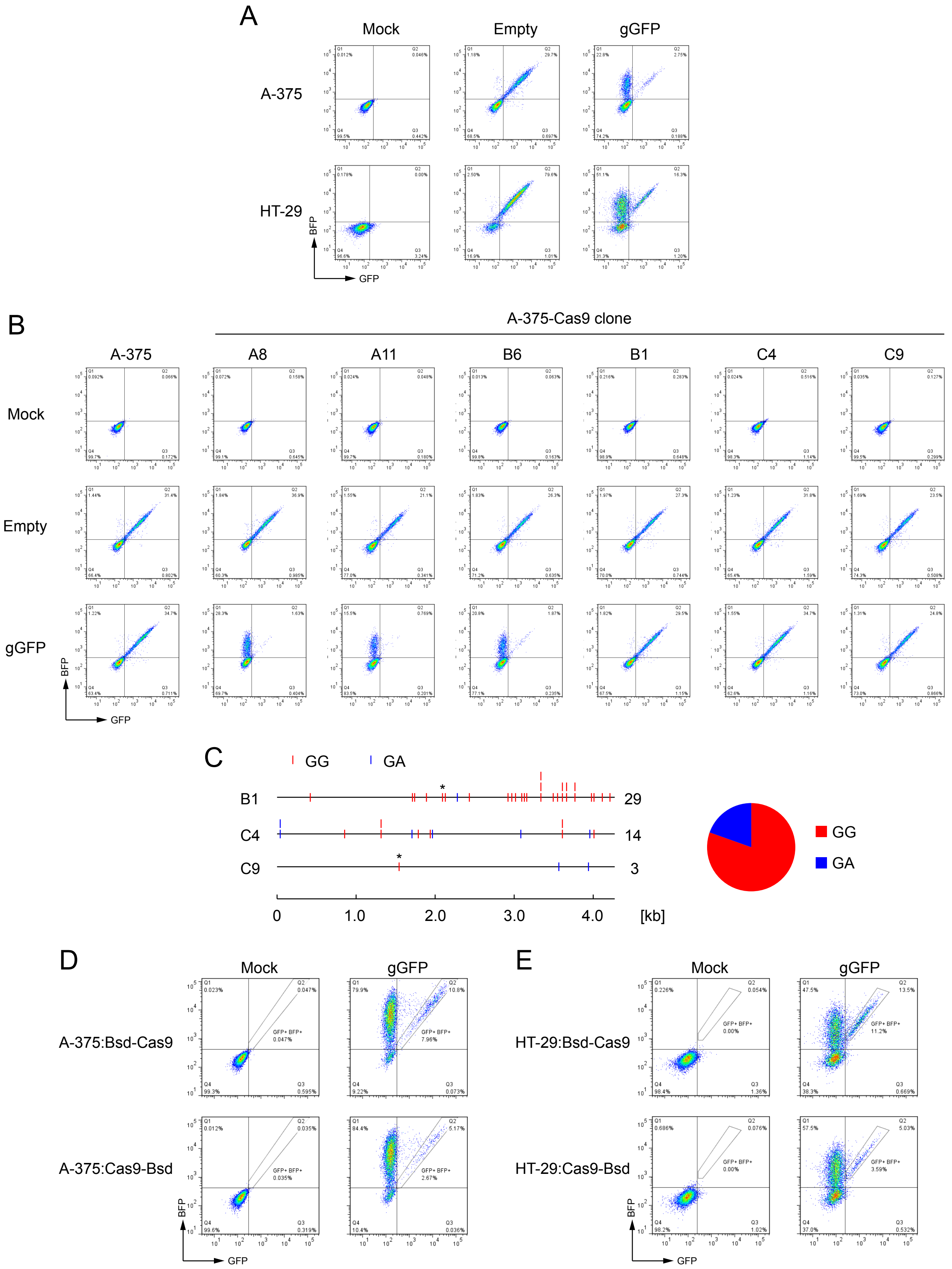
A
Mock
Empty
gGFP
A-375
HT-29
BFP
GFP
B
A-375-Cas9 clone
A-375
A8
A11
B6
B1
C4
C9
Mock
Empty
gGFP
BFP
GFP
C
GG
GA
B1
C4
C9
29
14
3
0
1.0
2.0
3.0
4.0
[kb]
GG
GA
D
Mock
gGFP
A-375:Bsd-Cas9
A-375:Cas9-Bsd
BFP
GFP
E
Mock
gGFP
HT-29:Bsd-Cas9
HT-29:Cas9-Bsd
BFP
GFP

Figure S2 (Related to Figure 2). **APOBEC3 signatures detected in the Cas9 coding sequence of Cas9-inactive cells.** **A**, Flow cytometry profiles of the Cas9 reporter assay in bulk A-375 (top) and HT-29 cells (bottom) expressing Cas9. A similar proportion of cells did not show Cas9 activity in both A-375 melanoma and HT-29 cells. **B**, Flow cytometry profiles of the Cas9 reporter assay in 6 A-375 subclones. While clones A8, A11 and B6 showed near-uniform Cas9 activity, clone B1, C4 and C9 had no Cas9 activity. **C**, Mutations detected in the Cas9-coding sequence in Clones B1, C4 and C9. Red and blue vertical lines represent mutations at the GG and GA context, respectively. The numbers on the right are the total number of mutations detected. Asterisks indicate nonsense mutations. A pie chart represents a proportion of each mutation signature. No mutations were detected from the Cas9-functional cell lines. **D, E**, Flow cytometry profiles of bulk A-375 (**D**) and HT-29 (**E**) cells harbouring Cas9 following (top panels) or followed by (bottom panels) the Blasticidin resistance gene. See also Figure S1C. The double-positive fractions were reduced by approximately 70% in both cell lines. The experiments were performed twice and the representative data were shown.

Figure S3

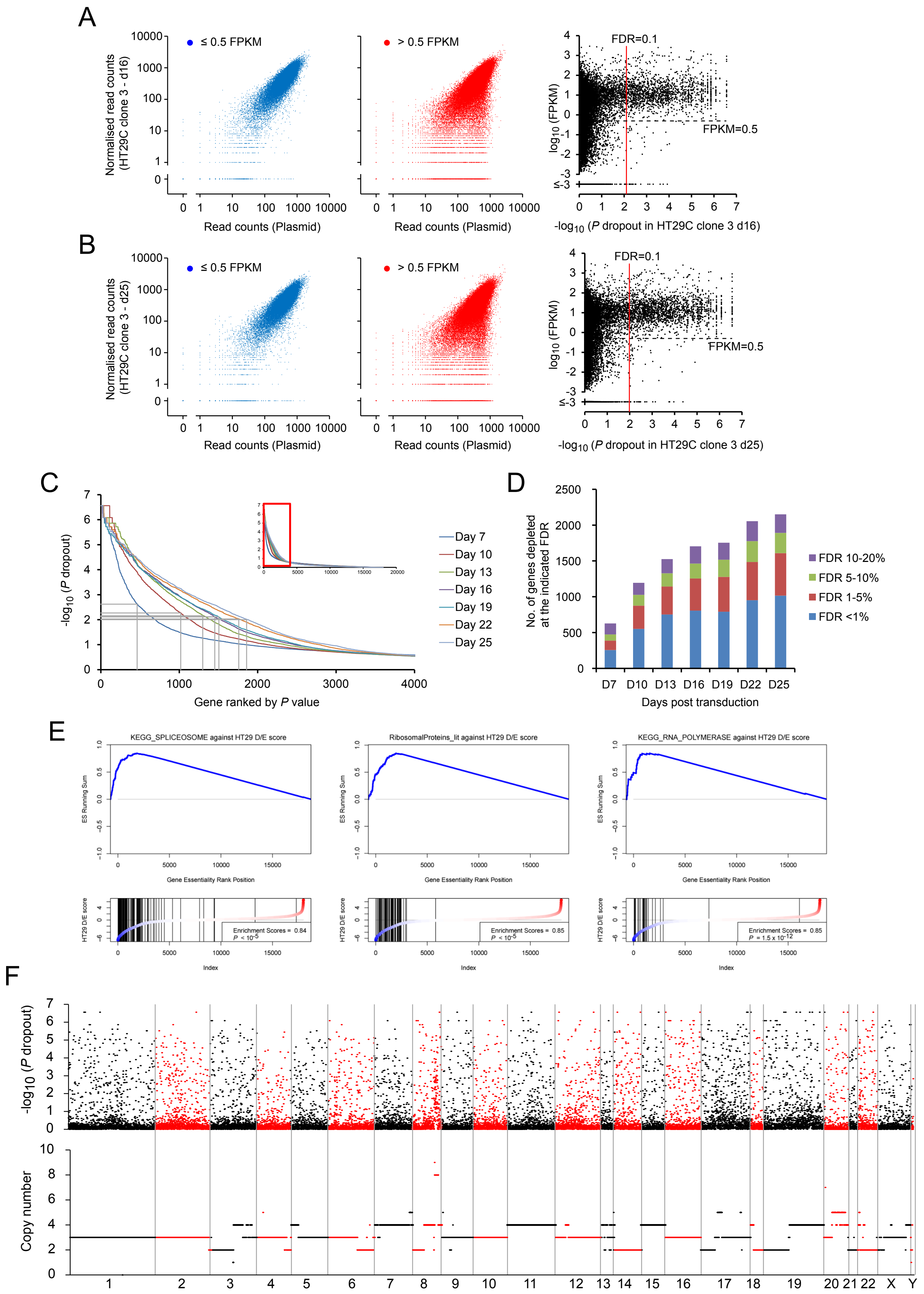
A
≤ 0.5 FPKM
> 0.5 FPKM
Normalised read counts (HT29C clone 3 - d16)
Read counts (Plasmid)
FDR=0.1
FPKM=0.5
log10 (FPKM)
-log10 (P dropout in HT29C clone 3 d16)
B
Normalised read counts (HT29C clone 3 - d25)
-log10 (P dropout in HT29C clone 3 d25)
C
-log10 (P dropout)
Gene ranked by P value
Day 7
Day 10
Day 13
Day 16
Day 19
Day 22
Day 25
D
No. of genes depleted at the indicated FDR
FDR 10-20%
FDR 5-10%
FDR 1-5%
FDR <1%
D7 D10 D13 D16 D19 D22 D25
Days post transduction
E
KEGG_SPLICEOSOME against HT29 D/E score
RibosomalProteins_lit against HT29 D/E score
KEGG_RNA_POLYMERASE against HT29 D/E score
ES Running Sum
Gene Essentiality Rank Position
HT29 D/E score
Enrichment Scores = 0.84
Enrichment Scores = 0.85
Enrichment Scores = 0.85
Index
F
-log10 (P dropout)
Copy number
1 2 3 4 5 6 7 8 9 10 11 12 13 14 15 16 17 18 19 20 21 22 X Y

Figure S3 (Related to Figure 2). **A CRISPR dropout screen in HT-29. A**, **B**, Comparison between gRNA counts (left and middle panel) or gene-level significance of dropout and gene expression at day 16 (**A**) and day 25 (**B**). RNA-seq data (GSE41586) were used for HT-29. **C**, A plot showing genes ranked by dropout *P* values from 7 time points. Grey horizontal lines indicate a statistical significance level at an FDR of 10% at each time point. **D**, A bar chart showing the number of genes depleted at the indicated statistical significance levels. **E**, Gene set enrichment analysis on spliceosome, cytoplasmic ribosome and RNA polymerase pathways as a dropout quality control assessment. Full results can be found in Supplementary Data 2. **F,** Genome-wide plots of depletion *P* values at day 25 and copy numbers.

Figure S4

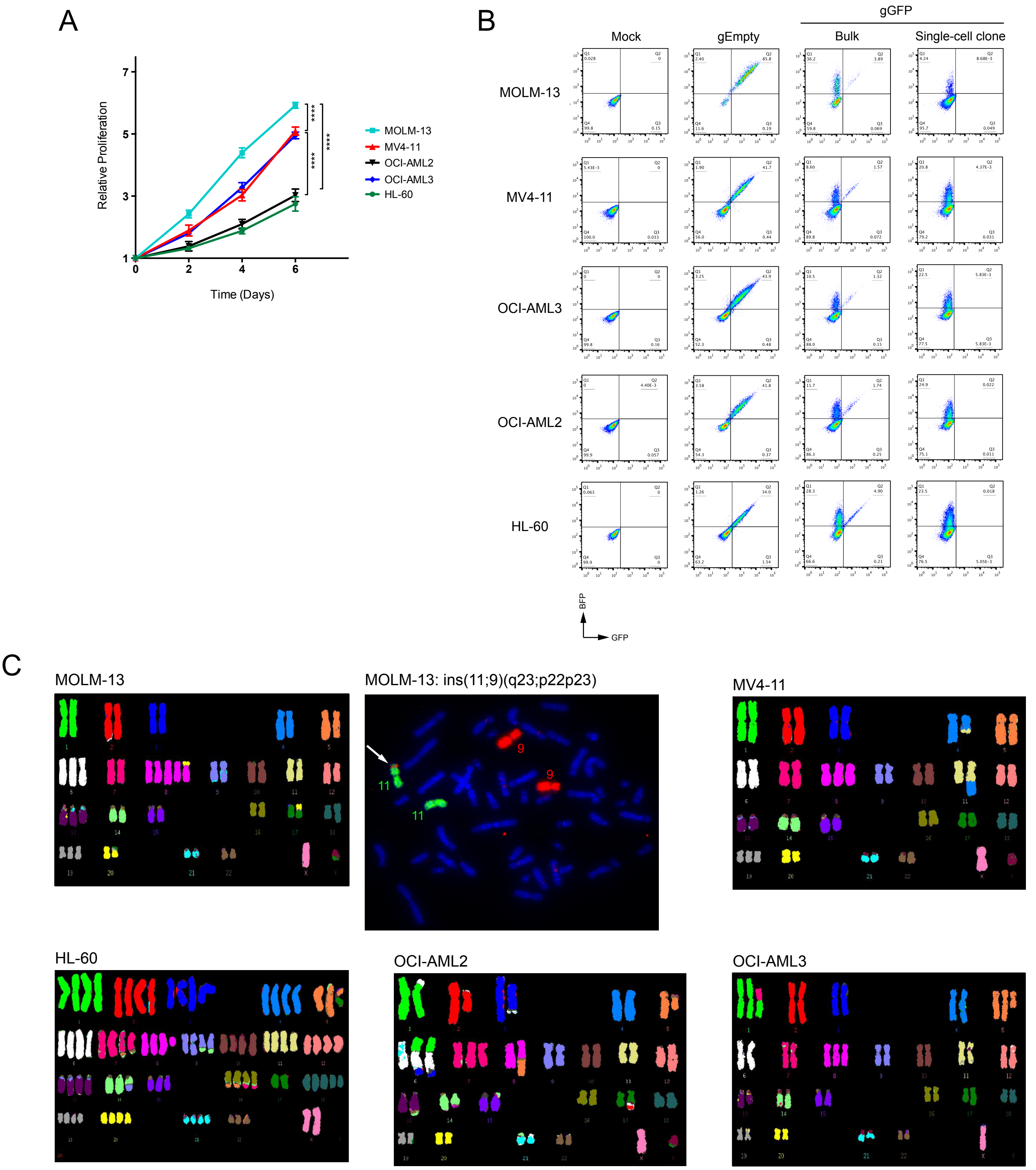

D

Cell line	Aberrations observed
MOLM-13	52,XY,+6,+8,+8,+der(8)t(8;20),ins(11;9)(q23;p22p23),+13,der(17)t(17;20),+19,der(20)t(17;20)
MV4-11	48,XY,t(4;11),+8,+19
HL-60	85,XX,-5,der(5)t(5;17),der(7)t(7;16)x2,r(8),der(9)t(9;14)x2,14,der(14)t(9;14),-15,der(16)t(5;16)x2, der(16)t(7;16)x2,-17,-17,+18,-22
OCI-AML2	47,XY,der(1)t(1;6),del(2),ins(3;2),der(3)t(1;3),del(5)t(5;8),+6,der(6)(1::6::3)x2,der(8)t(5;8), der(13)t(13;14),der(14)t(8;14),der(17)t(2;17)
OCI-AML3	48,X,+der(1)t(1;8)(p11;q11),+del(5q),+8

Figure S4 (Related to Figure 3). **Characterization of Cas9-expressing AML cell lines. A**, Proliferation rates of the 5 AML cell lines used. **B**, Cas9 functional assay in the AML cell lines. Note that double-positive cells (Cas9-inactive) were detected in each bulk population but not in single-cell clones. **C**, Chromosome paint FISH for each cell line. Chromosome translocation 11;9 in MOLM-13 was confirmed by chromosome FISH (arrow in the top-right panel). Translocation 4;11 in MV4-11 is visible on paint FISH. **D**, A summary of karyotype analysis.

Figure S5

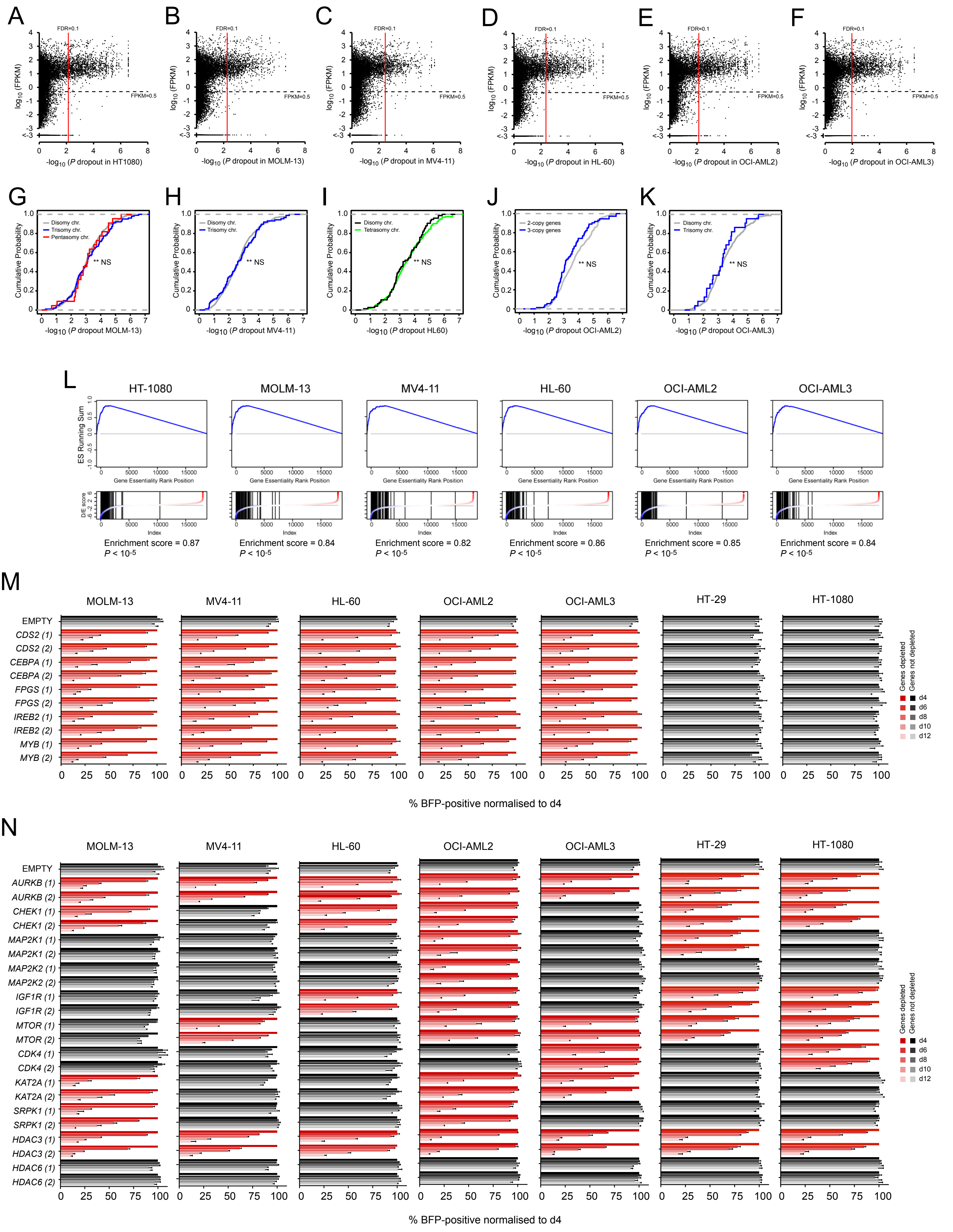
A
B
C
D
E
F
FDR=0.1
FPKM=0.5
log10 (FPKM)
-log10 (P dropout in HT1080)
-log10 (P dropout in MOLM-13)
-log10 (P dropout in MV4-11)
-log10 (P dropout in HL-60)
-log10 (P dropout in OCI-AML2)
-log10 (P dropout in OCI-AML3)
G
H
I
J
K
Cumulative Probability
Disomy chr.
Trisomy chr.
Pentasomy chr.
Tetrasomy chr.
2-copy genes
3-copy genes
** NS
-log10 (P dropout MOLM-13)
-log10 (P dropout MV4-11)
-log10 (P dropout HL60)
-log10 (P dropout OCI-AML2)
-log10 (P dropout OCI-AML3)
L
HT-1080
MOLM-13
MV4-11
HL-60
OCI-AML2
OCI-AML3
ES Running Sum
Gene Essentiality Rank Position
D/E score
Index
Enrichment score = 0.87
Enrichment score = 0.84
Enrichment score = 0.82
Enrichment score = 0.86
Enrichment score = 0.85
Enrichment score = 0.84
P < 10-5
M
MOLM-13
MV4-11
HL-60
OCI-AML2
OCI-AML3
HT-29
HT-1080
EMPTY
CDS2 (1)
CDS2 (2)
CEBPA (1)
CEBPA (2)
FPGS (1)
FPGS (2)
IREB2 (1)
IREB2 (2)
MYB (1)
MYB (2)
Genes depleted
Genes not depleted
d4
d6
d8
d10
d12
% BFP-positive normalised to d4
N
MOLM-13
MV4-11
HL-60
OCI-AML2
OCI-AML3
HT-29
HT-1080
EMPTY
AURKB (1)
AURKB (2)
CHEK1 (1)
CHEK1 (2)
MAP2K1 (1)
MAP2K1 (2)
MAP2K2 (1)
MAP2K2 (2)
IGF1R (1)
IGF1R (2)
MTOR (1)
MTOR (2)
CDK4 (1)
CDK4 (2)
KAT2A (1)
KAT2A (2)
SRPK1 (1)
SRPK1 (2)
HDAC3 (1)
HDAC3 (2)
HDAC6 (1)
HDAC6 (2)
Genes depleted
Genes not depleted
d4
d6
d8
d10
d12
% BFP-positive normalised to d4

Figure S5 (Related to Figures 3, 4 and 6). **CRISPR dropout screens in the 5 AML cell lines and HT-1080 fibrosarcoma cell line and the full result of the validation experiment. A-F**, Comparisons between dropout *P* values from indicated cell lines and their corresponding gene expression profile. RNA-seq data for HT-1080 were obtained from the ENCODE project. RNA-seq data for the AML cell lines were generated in this study. Note that the vast majority of depleted genes are expressed, indicating minimum off-target effects in these cell lines. **G-K**, Dropout efficiency of genes on aneuploid chromosomes in the AML cell lines indicated. Genes that belong to the common lethal gene class were plotted separately according to the number of residing chromosomes. Normality of the data was confirmed using quantile-quantile plot and thus Student's *t*-test was performed. No statistically significant difference was detected, indicating that copy number difference did not affect dropout efficiency in the cell lines studied. **L**, Gene set enrichment analysis on ribosomal protein genes as a dropout quality control assessment for the cell line indicated. Full results can be found in Supplementary Data 2. **M**, Genes depleted in all 5 AML cell lines tested. **N**, Genes selected for genetic and pharmacological validation (related to Fig. 3b). The experiment was performed using two guide RNAs per gene; one derived from our human CRISPR library (indicated as 1) and a new gRNA (indicated as 2). The gRNA sequences are listed in Table S6.

Figure S6

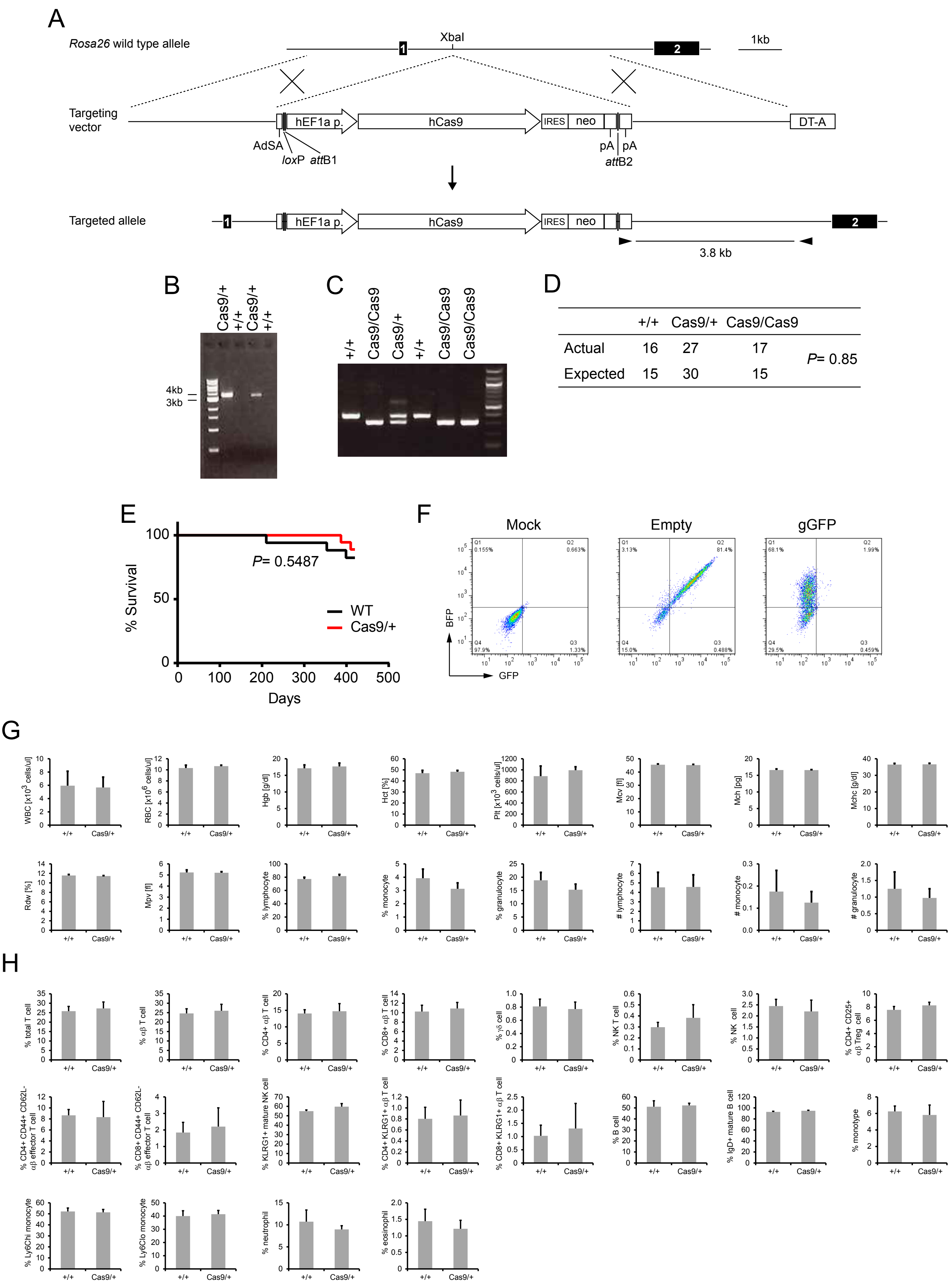
A
Rosa26 wild type allele
XbaI
1
2
1kb
Targeting vector
hEF1a p.
hCas9
IRES
neo
DT-A
AdSA
loxP
attB1
pA
pA
attB2
Targeted allele
3.8 kb
B
Cas9/+
+/+
Cas9/+
+/+
4kb
3kb
C
+/+
Cas9/Cas9
Cas9/+
+/+
Cas9/Cas9
Cas9/Cas9
D
+/+ Cas9/+ Cas9/Cas9
Actual 16 27 17
Expected 15 30 15
P= 0.85
E
% Survival
P= 0.5487
WT
Cas9/+
Days
F
Mock
Empty
gGFP
BFP
GFP
G
WBC [x10^3 cells/ul]
RBC [x10^6 cells/ul]
Hgb [g/dl]
Hct [%]
Plt [x10^3 cells/ul]
Mcv [fl]
Mch [pg]
Mchc [g/dl]
Rdw [%]
Mpv [fl]
% lymphocyte
% monocyte
% granulocyte
# lymphocyte
# monocyte
# granulocyte
+/+
Cas9/+
H
% total T cell
% αβ T cell
% CD4+ αβ T cell
% CD8+ αβ T cell
% γδ cell
% NK T cell
% NK cell
% CD4+ CD25+ αβ Treg cell
% CD4+ CD44+ CD62L- αβ effector T cell
% CD8+ CD44+ CD62L- αβ effector T cell
% KLRG1+ mature NK cell
% CD4+ KLRG1+ αβ T cell
% CD8+ KLRG1+ αβ T cell
% B cell
% IgD+ mature B cell
% monotype
% Ly6Chi monocyte
% Ly6Clo monocyte
% neutrophil
% eosinophil

Figure S6 (Related to Figure 5). **Generation and characterisation of a mouse line constitutively expressing Cas9. A**, Schematic depicting the gene targeting strategy. Black boxes, exons of the *Rosa26* gene; AdSA, adenovirus splice acceptor site; hEF1a p., intron-containing human elongation factor 1a promoter; hCas9, codon-optimised Cas9 with C-terminal NLS tag; IRES, internal ribosome entry site; neo, G418 resistant gene; pA, bovine growth hormone polyadenylation signal sequence; DT-A, diphtheria toxin A fragment expression cassette for negative selection; arrowhead, primers for the detection of homologous recombination. **B**, Long-range PCR screening of G418-resistant colonies. **C**, Genotyping PCR of mouse ear-clip lysates. **D**, The number of offspring with the indicated genotype from 7 litters. No statistically significant difference was detected by the X^2 test, indicating Mendelian inheritance of the *Cas9* allele. **E**, Survival analysis of Cas9 (n=18) and wild-type (n=17) mice. No statistically significant difference was detected by the log-rank test, indicating no organism-level toxicity of Cas9 expression in the course of observation. **F**, Cas9 functional assay in embryonic fibroblasts derived from $Rosa26^{Cas9/+}$ embryos. **G,H**, Haematological phenotyping of Cas9-expressing mice. For each genotype, 4 female mice were analysed. No statistically significant difference was detected by the Student's *t*-test with multiple comparison compensation. The data are shown as mean ± s.d.

Supplementary Tables

Table S1. Lists of gRNAs in the Mouse v2 and Human v1 CRISPR libraries (Related to Figures 1 and 2).

Table S2. Genes and enriched pathways in each cluster identified in the time-course HT-29 dropout experiment (Related to Figure 2).

Table S3 (Related to Figures 2 and 3). Statistical result on gene depletion in all human cancer cell lines used in this study. Depletion *P* values, depletion FDR and RNA-seq counts (log_{10}-transformed FPKM) are shown for each gene in each cell line. Note that genes whose RNA-seq count is equal to or less than 0.001 FPKM are all given a value of -3.5 as a log_{10}-transformed value. Summaries of depleted genes in each human cancer cell line at FDR 20% or 10% are also shown in separate spreadsheets.

Table S4. A list of "druggable" genes identified by DGIdb (Related to Figure 4).

Table S5. Human primary AML sample information (Related to Figure 7).

Table S6. Lists of gRNA and primer sequences used in this study (Related to Figures 4, 5 and 6).

Table S4. Genes in selected druggable categories (related to Figure 4)

AML-specific dropouts			Common dropouts
Clinically Actionable: 33 Genes	Histone Modification: 41Genes	Kinase: 26Genes	Clinically Actionable: 25 Genes
ARID1A	ATXN7L3	BRD2	ARFRP1
BBC3	BRMS1	BRD7	ATR
BCL2	BRPF1	BUB1B	AURKB
CBFB	CCDC101	CCNB1	CDK6
CEBPA	CCNB1	CCNH	CHEK1
DOT1L	CTR9	CLK2	CTCF
EP300	CXXC1	CSNK2A1	DICER1
ERCC1	DOT1L	DGKD	DNMT1
ERCC4	ENY2	EFNA3	ERCC2
FANCA	EP300	HIPK1	ERCC3
FANCD2	JMJD6	IPPK	MAX
FBXW7	KAT2A	ITPK1	MYC
HIST1H2AC	KAT6A	MAP2K2	NPM1
IRS2	KAT7	MASTL	NUP93
KAT6A	KDM2A	PIK3C2A	PDPK1
KMT2D	KMT2D	PIK3CG	POLE
KRAS	LDB1	PIM1	PPP2R1A
MAP2K2	MEN1	PRKAA1	RAD51
MCL1	MTA2	RFK	RAD51C
MEN1	PAF1	RPS6KA1	RAD51D
PIK3CG	PCGF1	SIK3	RHOA
PIM1	PRKAA1	SRPK1	RRM1
PRDM1	RING1	STRADA	SDHC
RAD51B	RNF168	TAF1	SETD2
RUNX1	RNF40	TBRG4	TOP2A
SDHAF2	RNF8	TRIM28	
SDHB	SETDB1		
SMARCB1	SIN3B		
SMARCD1	SIRT7		
STAG2	SPI1		
TERT	TADA2B		
TSC1	TAF1		
ZNF217	TAF12		
	TAF5		
	TAF5L		
	TAF6L		
	TBL1XR1		
	TCF3		
	UBE2N		
	WAC		
	WDR5		

Table S5. Human primary AML samples (related to Figure 7)

AML sample ID	Gender	Full Blood Count				Flow cytometry (bone marrow blasts)									Bone marrow karyotype	Bone Marrow FISH panel	Molecular genetic tests		
		WBC ($x10^9$/L)	Hb (g/L)	Plts ($x10^9$/L)	Blasts ($x10^9$/L)	CD34+ %	CD13+ %	CD33+ %	HLA DR+%	CD14 %	CD64 %	CD117 %	CD56 %	Aberrant markers			FLT3	NPM1	Other
AML1	F	8.3	109	129	5.39	98	9	99	95	3	0	98	0		Normal karyotype, 46 XX	Negative	WT	WT	
AML2	M	65.7	79	20	17.08	90	67	3	12	0	0	29	0	CD7 87%	Normal karyotype 46, XY	Negative	WT	WT	
AML3	F	10.2	121	59	1.93	0.4	93	98	1	0	97	71	57	CD9 63%	49,XX,+6,t(11;19),+13,+21[2]/50,XX,+6,add(10)(q 2?6), t(11;19),+13,+ider(19),+21[8],	MLL fusion 11q23	WT	WT	MLL-ENL (MLLT1) fusion
AML4	F	6.2	93	129	1.48	100	81	100	99	5	36	86	4		Complex caryotype del 5q, 7q, 10q and 12p (ETV6 by FISH), additional material 17, trisomy 11 and monosomy 16	Complex, ETV6 loss, MLL extra copy and TP53 deletion, del 5q and 7q	WT	WT	
AML5	F	21.4	119	58	16.05	2	92	99	91	0	33	91	0	CD7 44%, CD9 78%	Normal karyotype, 46 XX	Negative	WT	EXON12	
AML6	M	6.2	118	177	4.77	0	80	99	95	52	98		22		46,XY,add(8)(p?21)[9]/46,XY[11].nuc ish(5'MLLx3,3'MLLx2) (5'MLL con 3'MLLx1)[68/100]	68% POSITIVE for MLL (11q23) rearrangement, with an additional copy of the 5' (green) part of the probe	WT	WT	MLL partner not identified
AML7	M	133.7	8.3	52	117.65	97	99	5	78	0	3	97	0	CD9 78%	failed	Trisomy 8 (?partial)	WT	WT	
AML8	F	149.8	85	65	74.9	100	90	45	49	0	0	98	0	CD7 92%	Normal karyotype, 46XX	Negative	WT	WT	
AML9	F	13.9	105	20	10.29	24	31	97	56	0	4	99	0	CD7 89%	47XX, +X	Negative	WT	WT	
AML10	F	72.8	97	152	13.83	1	21	99	98	42	99	2	6	CD4 70%	Normal karyotype, 46 XX	Negative	ITD	EXON12	

Table S6. Sequences of guide RNAs used in this study

Gene	gRNA Sequence	gRNA ID from Human v1 library or coodinate (GRCh37)
CDK4(1)	GGTGGCTTTACTGAGGCGAC	CDK4_CCDS8953.1_ex6_12:58145310-58145333:-_5-5
CDK4(2)	ACCTCACGAACTGTGCTGAT	chr12:58,145,331-58,145,350
AURKB(1)	GAAAATAGTTGTAGAGACGC	AURKB_CCDS11134.1_ex3_17:8110171-8110194:+_5-3
AURKB(2)	GATGCTCTAATGTACTGCCA	chr17:8,109,920-8,109,939
KAT2A(1)	GGATGAGATAAACCGACTGC	KAT2A_CCDS11417.1_ex15_17:40272331-40272354:-_5-4
KAT2A(2)	CGGGGTGGGAGTCGGAATCG	chr17:40,273,191-40,273,210
CHEK1(1)	GTACTTACTGCAATGCTCGC	CHEK1_CCDS58191.1_ex4_11:125503221-125503244:+_5-4
CHEK1(2)	CGTTTGTTGAACAAGATGTG	chr11:125,503,117-125,503,136
SRPK1(1)	GGTGTGGATGATACGGCACT	SRPK1_CCDS47415.1_ex8_6:35840454-35840477:+_5-2
SRPK1(2)	CTGCATGGTATTTGAAGTTT	chr6:35,842,093-35,842,112
MTOR(1)	GACTTTTACCGCTGAGTACG	MTOR_CCDS127.1_ex54_1:11317057-11317080:-_5-5
MTOR(2)	AGCCTCATAGGAGTGGAAGG	chr1:11,317,201-11,317,220
MAP2K1(1)	GATGGTGCGTTCTACAGCGA	MAP2K1_CCDS10216.1_ex2_15:66729179-66729202:+_5-3
MAP2K1(2)	TGGAGATCAAACCCGCAATC	chr15:66,729,094-66,729,113
MAP2K2(1)	GGCAACTCGCCGTACATCGT	MAP2K2_CCDS12120.1_ex8_19:4110561-4110584:-_5-4
MAP2K2(2)	CTCTTTCAGCACCTGGTCCA	chr19:4,102,419-4,102,438
IGF1R(1)	GATGATGCGATTCTTCGACG	IGF1R_CCDS10378.1_ex6_15:99454576-99454599:-_5-4
IGF1R(2)	TTCAGAGCTGGAGAACTTCA	chr15:99,442,716-99,442,735
HDAC3(1)	GGTAATGCAGGACCAGGCTA	HDAC3_CCDS4264.1_ex13_5:141016134-141016157:+_5-5
HDAC3(2)	AGAGACCGTAATGCAGGACC	chr5:141,016,129-141,016,148
HDAC6(1)	GCAGTGCTACAGTCTCGCAC	HDAC6_CCDS14306.1_ex15_X:48674388-48674411:+_5-4
HDAC6(2)	GATGATCCGCAAGATGCGCT	chrX:48,674,568-48,674,586
CDS2 (1)	GACCCCGGAGGTCCTCAATA	CDS2_CCDS13088.1_ex1_20:5154260-5154283:+_5-1
CDS2 (2)	GCGATTATCATCAAAACCAT	CDS2_CCDS13088.1_ex2_20:5155904-5155927:-_5-2
CEBPA (1)	GCTGGCCGCAGTGCGCGATC	CEBPA_CCDS54243.1_ex0_19:33792674-33792697:+_5-1
CEBPA (2)	GCCCCGACGCGCTCGTACAG	CEBPA_CCDS54243.1_ex0_19:33792865-33792888:+_5-2
FPGS (1)	GGACGGGATTCTTTAGGTAC	FPGS_CCDS35148.1_ex3_9:130566963-130566986:+_5-2
FPGS (2)	GGGGAGCGGATCCGCATCAA	FPGS_CCDS35148.1_ex4_9:130569273-130569296:+_5-3
IREB2 (1)	GGTTCTGCCTTACTCAATAC	IREB2_CCDS10302.1_ex2_15:78755264-78755287:+_5-1
IREB2 (2)	GGAGAACTAGGCCGAAACTC	IREB2_CCDS10302.1_ex4_15:78758742-78758765:+_5-3
MYB (1)	GGAAATACGGTCCGAAACGT	MYB_CCDS47481.1_ex4_6:135511280-135511303:+_5-1
MYB (2)	GATGCGTCGGAAGGTCGAAC	MYB_CCDS47481.1_ex5_6:135513497-135513520:+_5-3

Table S6 *(continued)*

Gene	gRNA Sequence	gRNA ID from the mouse v2 library or coodinate (GRCm38)
Aurkb(1)	GAAGAAGAGCCGTTTCATCG	Aurkb_CCDS24877.1_ex3_11:69048255-69048277:+_5-2
Aurkb(2)	TTTCGATCTCTCGGCGAAGC	chr11:69,048,330-69,048,349
Kat2a(1)	TGTCCCCTCCGAAGGTGGCA	chr11:100,709,402-100,709,421
Kat2a(2)	AAGGCTTCGGCCAAACACGT	chr11:100,710,555-100,710,574
Srpk1(1)	ACCTGCAGACCCCGATGGTG	chr17:28,602,686-28,602,705
Srpk1(2)	TGAATGAGCAGTACATTCGA	chr17:28,602,752-28,602,771
Chek1(1)	GCTGTCAGGAATATTCTGAT	chr9:36,718,389-36,718,408
Chek1(2)	TGCAGTAAGTACTATTCCAC	chr9:36,719,517-36,719,536
Piga (1)	TCTCAGTGCCTCATTGAGAG	chrX:164,422,814-164,422,833
Piga (2)	CCTCATTGAGAGAGGGCACA	chrX:164,422,822-164,422,841
Piga (3)	ATAACTGTCACCCATGCTTA	chrX:164,422,847-164,422,866
Piga (4)	CCATGCTTATGGAAATCGAA	chrX:164,422,858-164,422,877
Piga (5)	GGCGTCCGTTACCTCACCAA	chrX:164,422,880-164,422,899
Piga (6)	TTCACAGTCTGCCATTGCTC	chrX:164,422,965-164,422,984
Piga (7)	GCTCAGGTACATATTTGTTC	chrX:164,422,981-164,423,000
Piga (8)	CCACAGTTCTTTCTCTGCCA	chrX:164,423,026-164,423,045
Piga (9)	TCTCTTCCACGCCAAGACAA	chrX:164,423,059-164,423,078
Piga (10)	CGGATTTGCTGATGTCAGCT	chrX:164,423,116-164,423,135
Piga (11)	TCACTCCAGACCCATTTAGG	chrX:164,423,289-164,423,308
Piga (12)	AGCAGACTTGTTTACAGAAA	chrX:164,423,342-164,423,361
Piga (13)	CAAGAATTACATTTCCTAAT	chrX:164,427,979-164,427,998
Piga (14)	GAATTACATTTCCTAATTGG	chrX:164,427,982-164,428,001
Piga (15)	ACATTTCCTAATTGGAGGAG	chrX:164,427,987-164,428,006
Piga (16)	CATTTCCTAATTGGAGGAGA	chrX:164,427,988-164,428,007
Piga (17)	GAATCATTTTGGAAGAAGTA	chrX:164,428,019-164,428,038
Piga (18)	AAAGATACCAACTACATGAC	chrX:164,428,043-164,428,062
Piga (19)	AGCGTTCTGCATGGCCATCG	chrX:164,428,627-164,428,646
Piga (20)	ATCGTGGAAGCTGCCAGTTG	chrX:164,428,643-164,428,662
Piga (21)	AGCTGCCAGTTGTGGTTTGC	chrX:164,428,651-164,428,670
Piga (22)	TCACTCCAGACCCATTTAGG	chrX:164,423,289-164,423,308
Piga (23)	GAAGAGAGCATCATGGGCCA	chrX:164,423,046-164,423,065

Supplementary Datasets

Supplementary Dataset 1. Mouse CRISPR screen data (Related to Figure 1)

1. Raw gRNA counts in mouse ES cells with the v1 library
2. Raw gRNA counts in mouse ES cells with the v2 library
3. Gene-level MAGeCK output for mouse ES cells with the v1 library
4. Gene-level MAGeCK output for mouse ES cells with the v2 library
5. RNA-seq data for mouse ES cells (GSE44067)

Supplementary Dataset 2. Human CRISPR screen data (Related to Figures 2 and 3)

1. Raw gRNA counts in 5 AML cell lines
2. Raw gRNA counts in HT-29 (time course, d7-d25) and HT-1080
3. Gene set enrichment analysis as quality check of the screens
4. Gene-level MAGeCK output for MOLM-13
5. Gene-level MAGeCK output for MV4-11
6. Gene-level MAGeCK output for HL-60
7. Gene-level MAGeCK output for OCI-AML2
8. Gene-level MAGeCK output for OCI-AML3
9. Gene-level MAGeCK output for HT-1080
10. Gene-level MAGeCK output for HT-29 at day 7
11. Gene-level MAGeCK output for HT-29 at day 10
12. Gene-level MAGeCK output for HT-29 at day 13
13. Gene-level MAGeCK output for HT-29 at day 16
14. Gene-level MAGeCK output for HT-29 at day 19
15. Gene-level MAGeCK output for HT-29 at day 22
16. Gene-level MAGeCK output for HT-29 at day 25
17. RNA-seq data for all 7 cell lines studied

Supplementary Experimental Procedures

Plasmid construction

All plasmids but gene-specific gRNA vectors have been deposited with Addgene (67974-67991, 68343, 72666 and 72667). Mouse v2 (67988) and Human v1 (67989) libraries are also available from Addgene. gRNA sequences used in this study can be found in Table S6.

A lentiviral backbone vector, pKLV2, was first constructed by assembling gBlock fragments (IDT) into pBluescriptIIKS+ using Gibson assembly master mix (NEB). The U6gRNA3(BbsI) and U6gRNA5(BbsI) fragments, which carry the conventional(Mali et al., 2013) and the improved(Chen et al., 2013) gRNA scaffold, were synthesized as gBlock fragments and cloned into the MluI-BamHI site of pKLV2, resulting in pKLV2-U6gRNA3(BbsI)-PGKpuro2ABFP and pKLV2-U6gRNA5(BbsI)-PGKpuro2ABFP, respectively. The Woodchuck Hepatitis virus posttranscriptional regulatory element (WPRE) was synthesized as a gBlock fragment with the BbsI site in the element mutated and cloned into the NotI-KpnI site of pKLV2-U6gRNA5(BbsI)-PGKpuro2ABFP, resulting in pKLV2-U6gRNA5(BbsI)-PGKpuro2ABFP-W. Subsequently, BFP was replaced with ZsGreen (ZsG), mAzamiGreen (mAG) or mCherry, resulting in pKLV2-U6gRNA5(BbsI)-PGKpuro2AZsG/mAG/mCherry-W, respectively.

To construct a dual gRNA expression vector, the SapI sites in pKLV2-U6gRNA5(BbsI)-PGKpuro2ABFP-W were mutated by site-directed mutagenesis, resulting in pKLV2.2-U6gRNA5(BbsI)-PGKpuro2ABFP-W. One of the 2 sites was within the lentiviral backbone and no effect on lentiviral production and transduction efficiency was confirmed. The h7SKgRNA5(SapI) and mU6gRNA5(SapI) fragments were synthesized as a gBlock fragment and cloned into the MluI site, resulting in pKLV2.2- h7SKgRNA5(SapI)-U6gRNA5(BbsI)-PGKpuro2ABFP-W and pKLV2.2- mU6gRNA5(SapI)-U6gRNA5(BbsI)-PGKpuro2ABFP-W, respectively.

The Cas9-expressing lentiviral vectors were constructed as follows. The Cas9 coding sequence (Cong et al., 2013) was synthesized as gBlock fragments and assembled into pBluescriptIIKS+, resulting in pBS-Cas9. The Bsd2A and 2ABsd fragments were then cloned into the N- and C- terminus of pBS-Cas9, resulting in pBS-Bsd2ACas9 and pBS-Cas92ABsd, respectively. Finally, the AscI-NotI fragment containing Bsd-fused Cas9 was cloned into the AscI-NotI site of pKLV2-EF1a-W, which was constructed by cloning human EF1a promoter and WPRE into pKLV2.

The Cas9 reporter vectors were constructed as follows. The PCR-generated BFP2AGFP fragment was used to replace the puro2ABFP portion of pKLV2-U6gRNA5(BbsI)-PGKpuro2ABFP-W, resulting in pKLV2-U6gRNA(Empty)-PGKBFP2AGFP-W. A gRNA targeting GFP (gGFP), GGGCGAGGAGCTGTTCACCG, was cloned into the BbsI site, resulting in pKLV2-U6gRNA(gGFP)-PKGBFP2AGFP-W. Subsequently, the BFP portion of the empty and the gGFP-expressing vector was replaced with mCherry, resulting in pKLV2-U6gRNA(Empty or gGFP)-PGKmCherry2AGFP-W. Alternatively, the GFP2ABFP fragment (the BFP coding sequence was mutated to create new PAM sequences) was used to generate pKLV2-U6gRNA(Empty)-PGKGFP2ABFP-W. A gRNA targeting BFP (gBFP), GAGCACGCCCCCGTCCTCGT, was cloned into the BbsI site, resulting in pKVL2-U6gRNA(gBFP)-PGKGFP2ABFP-W. Finally, the GFP portion of the vectors were replaced with mCherry, resulting in pKLV2-U6gRNA(Empty or gBFP)-PGKmCherry2ABFP-W.

The Rosa26 targeting vector carrying the Cas9 expression cassette was constructed as follows. pENTR-2B (Invitrogen) was first modified by cloning the PCR-generated GFP fragment carrying the BamHI-MluI and the SpeI-XhoI site at the 5’ and the 3’ end, respectively, into the BamHI-XhoI site, resulting in pENTR-GFP. The EF1a-Cas9 fragment (the MluI-NotI fragment of pEF1a-Cas9), PCR-generated bovine growth hormone polyadenylation signal sequence (bpA; the NotI-BsiWI and the SpeI site at the 5’ and the 3’ end, respectively) were cloned into the MluI-SpeI site of pENTR-GFP, resulting in pENTR-EF1aCas9bpA. The PCR-generated IRES-

neo was then cloned into the NotI-BsiWI site of pENTR-EF1aCas9bpA, resulting in pENTR-EF1aCas9IRESneobpA. Finally, the EF1aCas9IRESneobpA cassette was transferred by Gateway cloning (Invitrogen) to the Rosa26 targeting vector carrying the Gateway cloning site, resulting in pRosa26-EF1a-hCas9IRESneo.

Genome-wide guide RNA design

Genome-wide gRNAs were designed with a new design pipeline as follows. CCDS transcript sets were used as a basis for designing gRNAs targeting coding regions (mouse, release 13: 05/08/2013 on the GRCm38; human, release 15: 29/11/2013 on the GRCh37). Only CCDS records labeled as 'Public' were considered. The gRNA libraries were designed through the following four steps: i) identification of all possible gRNA target sites, ii) removal of unwanted gRNAs, iii) computation of design scores, and iv) selection of gRNAs.

i) Identification of all possible gRNA target sites. All gRNAs predicted to induce DSBs (assumed to be generated between the fourth and the third nucleotide upstream the PAM) within any CCDS exons were collected. Note that in some cases this can happen when part of the guide sequence or PAM aligns to the flanking introns.

ii) Removal of unwanted gRNAs. At this stage, gRNAs that contain BbsI sites or RNA polIII terminator sequences (a stretch of 5 Ts) were removed.

iii) Computation of design scores. The following scores were computed for each gRNA.

1. Whether the gRNA aligns to an off-target exonic site with up to one mismatch in the seed region (12bp upstream of the PAM), for maximum stringency. When searching for off-target exonic hits, a merged dataset of transcripts consisting of CCDS 'public' transcripts and RefSeq transcripts was used. RefSeq transcripts were downloaded from the UCSC table browser.
2. Whether the gRNA aligns to an off-target exonic site with up to two mismatches in the seed region (12bp upstream of the PAM). The same merged dataset of transcripts consisting of CCDS 'public' transcripts and RefSeq transcripts was used.
3. The number of off-target genomic matches with up to three mismatches
4. The number of off-target genomic matches with exactly four mismatches
5. (Human gRNA library only) Variant allele position and population frequency of any overlapping 1000 Genomes Project SNVs or indels.
6. The number of thymidine in the last 5 nucleotides of gRNAs. If there is 0 or 1 T anywhere in this region, the scores of 0 or 1 were given, respectively. If there are 2 Ts at the 4th and 5th positions from the PAM, the score of 2 was given. Otherwise, the score of 3 was given.

iv) Selection of gRNAs. As many as possible but up to five gRNAs were chosen for each CCDS transcript. gRNAs were selected transcript by transcript. However, if a selected gRNA also cut another CCDS transcript of the same gene, this overlap was noted and taken into account later when gRNAs were searched for the latter transcript(s). In this way, it was possible that five gRNAs chosen for the first transcript would also cut all other CCDS transcripts of the same gene. In addition, already chosen gRNAs would constrain the placement of new gRNA candidates in that new gRNAs were only allowed to overlap with previous gRNAs over a set number of nucleotides at most (see below). A strategy involving sets of gRNA selection criteria cascading from more stringent to more lenient was used to prioritize gRNAs. The sets of rules are depicted in the tables below flowing from rule set I to IV and numerically within each rule set. For example, at the initial stage rules in I-1 were in effect, so only gRNA candidates matching all the conditions in I-1 were considered. This subset of gRNAs was used in choosing as many gRNAs for each transcript as possible. At the next stage (I-2), the rule on how many nucleotides new gRNAs are allowed to overlap with previously chosen ones is relaxed from 5bp to

10bp. Using rule set I-2, more gRNAs will then be chosen for transcripts that still have fewer than five assigned gRNAs.

The gRNAs made available at each rule set were prioritized per transcript using the score (#genomic hits with ≤3)x100+(#genomic hits with 4 mismatches). For each transcript, gRNAs were then assigned in order from lowest to highest score until gRNA candidates ran out or until five gRNAs were assigned to the transcript. The process was repeated until rule set VI-6, at which point all 'possible' gRNAs have been chosen but some transcripts/genes may still have fewer than five gRNAs assigned.

Cascading rule I	1	2	3	4	5	6
Cut each transcript of gene	Yes					
gRNAs per transcript	5					
Exclude OT exonic hits with up to *x* seed MMs	2					
Cut transcripts *x* bp after ATG	100				80	80
Cut within the first *x* % of CDS	50		60	60	70	70
Max. gRNA overlap (bp)	5	10		10		10
Max. SNP allele frequency	0.01					
Max. score of trailing T	1					

Cascading rule II	1	2	3	4	5	6
Cut each transcript of gene	Yes					
gRNAs per transcript	5					
Exclude OT exonic hits with up to *x* seed MMs	1					
Cut transcripts *x* bp after ATG	100				80	80
Cut within the first *x* % of CDS	50		60	60	70	70
Max. gRNA overlap (bp)	5	10		10		10
Max. SNP allele frequency	0.01					
Max. score of trailing T	2					

Cascading rule III	1	2	3	4	5	6
Cut each transcript of gene	No					
gRNAs per transcript	5					
Exclude OT exonic hits with up to *x* seed MMs	2					
Cut transcripts *x* bp after ATG	100				80	80
Cut within the first *x* % of CDS	50		60	60	70	70
Max. gRNA overlap (bp)	5	10		10		10
Max. SNP allele frequency	0.01					
Max. score of trailing T	1					

Cascading rule IV	1	2	3	4	5	6
Cut each transcript of gene	No					
gRNAs per transcript	5					
Exclude OT exonic hits with up to *x* seed MMs	1					
Cut transcripts *x* bp after ATG	100				80	80
Cut within the first *x* % of CDS	50		60	60	70	70
Max. gRNA overlap (bp)	5	10		10		10
Max. SNP allele frequency	0.01					
Max. score of trailing T	1					

Cascading rule V	1	2	3	4	5	6
Cut each transcript of gene	Yes					
gRNAs per transcript	5					
Exclude OT exonic hits with up to *x* seed MMs	0					
Cut transcripts *x* bp after ATG	100				80	80
Cut within the first *x* % of CDS	50		60	60	70	70
Max. gRNA overlap (bp)	5	10		10		10
Max. SNP allele frequency	0.01					
Max. score of trailing T	2					

Cascading rule VI	1	2	3	4	5	6
Cut each transcript of gene	No					
gRNAs per transcript	5					
Exclude OT exonic hits with up to *x* seed MMs	0					
Cut transcripts *x* bp after ATG	100				80	80
Cut within the first *x* % of CDS	50		60	60	70	70
Max. gRNA overlap (bp)	5	10		10		10
Max. SNP allele frequency	0.01					
Max. score of trailing T	3					

From the initial list generated by the design pipeline, gRNAs that target olfactory receptor genes, or more than either 1 exonic or 3 genome-wide off-target sites with perfect guide sequence match were removed, resulting in Mouse v2 library consisting of 90,230 guide sequences targeting a total of 18,424 mouse genes and Human v1 library consisting of 90,709 guide sequences targeting a total of 18,010 human genes (Table S1).

Lentiviral gRNA library construction

Libraries were constructed as described before(Koike-Yusa et al., 2014) with a minor modification. pKLV2-U6gRNA5(BbsI)-PGKpuro2ABFP-W was used. Since the new lentiviral gRNA expression vector produces different 5’ overhangs after BbsI digestion, pooled oligos were synthesized with the following sequence: 5’-GCAGATGGCTCTTTGTCCTAGACATCGAAGACAACACCGN_{19}GTTTTAGTCTTCTCGTCGC-3’, where N_{19} represent guide sequences.

Cell culture

JM8.F6 mouse ESCs(Pettitt et al., 2009) and 293FT (Invitrogen) were cultured as described previously(Koike-Yusa et al., 2014). HT-29 was cultured in DMEM (Invitrogen) supplemented with 10% FBS (Invitrogen) and 1% GlutaMax (Invitrogen). A-375 was cultured in RPMI (Invitorgen) supplemented with 10% FBS (Invitrogen), 2 mM L-glutamine, 1 mM sodium pyruvate, 25 mM HEPES and toped up glucose to the final concentration of 4.5 g L^{-1}. HT-1080 was cultured in EMEM (Invitrogen) supplemented with 20% FBS (PAA) and 1% penicillin/streptomycin/glutamine (Invitrogen). MOLM-13, MV4-11 and HL-60 were cultured in RPMI1640 (Invitrogen) supplemented with 10% FBS (PAA) and 1% penicillin/streptomycin/glutamine. OCI-AML2 and OCI-AML3 were cultured in alpha-MEM (Lonza) supplemented with 20% FBS (PAA) and 1% penicillin/streptomycin/glutamine. HPC-7 was cultured in IMDM (Invitrogen) supplemented with 10% FBS, 100ng ml^{-1} SCF (Peprotech), 7.48 x 10^{-5} M 1-thioglycerol (Sigma), 1% penicillin/streptomycin/glutamine. All cancer cell lines were obtained from the Sanger Institute Cancer Cell Collection and negative for mycoplasma contamination.

Lentivirus production and transduction

Lentiviruses were produced as described previously (Koike-Yusa et al., 2014) for the AML cell lines and HT-1080 cells. For mouse ESCs and HT-29, packaging plasmids, psPax2 and pMD2.G (Addgene) were used at the following mixing ratio: 5.4 µg lentiviral vector, 5.4 µg psPax2 and 1.2 µg pMD2.G per 10-cm dish. Transduction of all human and primary mouse AML cells was performed in 6-well plates as follows: 1 x 10^6 cells and viral supernatant were mixed in 2 ml of culture medium supplemented with 8 µg ml^{-1} (human) or 4 µg ml^{-1} (mouse) polybrene (Millipore), followed by spinfenction (90 min, 900 *g*, 32 °C) and further incubated overnight at 37 °C. The medium was refreshed on the following day and the transduced cells were cultured further.

Generation of Cas9-expressing cancer cell lines

All Cas9-expressing cancer cell lines for screening were transduced with a virus produced from pKLV2-EF1aBsd2ACas9-W. Blasticidin selection was initiated 3 days after transduction at 10 µg ml^{-1} for all AML cell lines and HT-1080 or 20 µg ml^{-1} for HT-29 and A-375. After stable cell lines were established, the transduced cells were single-cell sorted into 96-well plates (MoFlo XDP). Clonally derived lines were further expanded and analysed by the Cas9 reporter lentiviruses.

Cas9 functional assay

Cells were transduced with a lentivirus produced with pKLV2-U6gRNA5(gGFP)-PGKBFP2AGFP-W vector as described above. As a negative control, pKLV2-U6gRNA5(Empty)-PGKBFP2AGFP-W lentiviral vector was used. The ratio of BFP only and GFP-BFP-double positive cells were analysed on a BD LSRFortessa instrument (BD) 3-4 days post transduction for mouse ESCs and adherent cancer cells or 8 days post transduction for AML cell lines. The data were subsequently analyzed using FlowJo.

Generation of genome-wide mutant libraries and screening

3.0 × 10^7 cells were transduced with a pre-determined volume of the genome-wide gRNA lentiviral supernatant that gave rise to 30% transduction efficiency measured by BFP expression. Two independent infections were conducted per cell line for the AML cell lines and HT-1080. HT-29 was transduced in triplicate. Two days after transduction, BFP expression were analysed by flow cytometry and cultures that showed 25-35% BFP-positive were selected with puromycin at 0.7 µg ml^{-1} (the AML cell line and HT-1080) or 1.5 µg ml^{-1} (HT-29 and mouse ESCs) for 4 days and further cultured. At every passage, 5.0 x 10^7 cells were seeded in new tissue culture plates. Approximately 1 x 10^8 cells of mouse ESCs were harvested 14 days post transduction. For HT-29, approximately 1 x 10^8 cells were harvested every 3 days between day 7 and day 25 post transduction. The AML cell lines and HT-1080 were harvested on day 25 post transduction.

Illumina sequencing of gRNAs and statistical analysis

Genomic DNA extraction and Illumina sequencing of gRNAs were conducted as described previously (Koike-Yusa et al., 2014). For HT-29C clone 3 and HT-1080, 19-bp single-end sequencing was performed with the custom sequencing primer 5'-TCTTCCGATCTCTTGTGGAAAGGACGAAACACCG-3'. The numbers of reads for each guide were counted with an in-house script. Enrichment and depletion of guides and genes were analysed using MAGeCK statistical package (Li et al., 2014) by comparing read counts from each cell line with counts from matching plasmid as the initial population.

gRNA competitive proliferation assay

gRNA competition assays were performed using pKLV2-U6gRNA5(BbsI)-PGKpuro2ABFP-W or pKLV2-U6gRNA5(BbsI)-PGKBsd2ABFP. Validation of dual target genes (*MAP2K1* and *MAP2K2*) was performed by using pKLV2-h7SKgRNA(BbsI)-U6gRNA5(BbsI)-PGKpuro2ABFP-W. For the validation of individual target genes, one gRNA was derived from the CRISPR library used in the screens and another gRNA was designed using

http://www.sanger.ac.uk/htgt/wge/. Viral supernatants were collected 48 h after transfection. All transfections and viral collections were performed in 24-well plates and transduction was performed as mentioned above. For gRNA/BFP competition assays, flow cytometry analysis was performed on 96-well plates using a LSRFortessa instrument (BD). Gating was performed on live cells using forward and side scatter, before measuring of BFP^+ cells.

Drug and proliferation assays

3 × 10^4 human or primary mouse cells were plated onto 96-well plates in a volume of 100 µl per well with vehicle or the indicated concentrations of Barasertib (0.04-10 µM, Selleckchem), AZD7762 (0.04-5 µM, Selleckchem), MK8776 (0.04-5 µM, Selleckchem), Trametinib (0.0008-100 nM, Selleckchem) , PQ401 (0.2-25 µM, Selleckchem) and MB-3(0.78-500 µM, Abcam). Plates were measured 72 h post-treatment. All the compounds were dissolved in DMSO. For measuring the proliferation of the human or primary mouse cells, 1 × 10^4 cells were plated onto 96-well plates in a volume of 100 µl and plates measured every 48 h, for 3 timepoints. CellTiter 96 AQueous Non-Radioactive Cell Proliferation Assay (Promega) was used for both assays.

Adult primary leukaemia and cord blood sample analysis

All human AML and cord blood samples were obtained with informed consent under local ethical approval (REC 07-MRE05-44). AML patient bone marrow and peripheral blood samples were processed as soon as possible after collection; mononuclear cells (MNC) were obtained by Ficoll gradient centrifugation, red blood-cell lysed and frozen immediately. An aliquot of up to 80000 fresh MNC was cultured in methylcellulose-based medium with multi-lineage cytokines (H4435, Stem Cell Technologies) for 7 days to identify samples with medium-to-high colony-forming capacity. Pre-tested samples were thawed into IMDM 10%FCS and tested for colony-forming efficiency in H4435 semi-solid medium (Stem Cell Technologies) that had been pre-mixed with MB3 or DMSO (vehicle, final concentration 0.2%) prior to addition of the cells. Colonies were quantified by microscopy 10-11 days after plating. Cord blood samples were processed within 24 hours of collection and kept at room temperature with mild agitation or at 4DEGC prior to processing. The MNC fraction was obtained as described above and enriched for stem and progenitor cells using human CD34 MicroBead kit (Miltenyi Biotec) or EasySep Progenitor Enrichment kit with Platelet Depletion (Stem Cell Technologies). CD34-enriched cells were immediately plated on H4435 multi-lineage methylcellulose medium in the presence of MB-3 or vehicle, as described above. Colonies were counted after 12-14 days.

RNA-seq analysis

For the AML cell lines, 5 × 10^5 cells were harvested and total RNA was purified using Arcturus Picopure RNA Isolation Kit (Invitrogen) according to the manufacturer's instructions. Two independent extractions were performed for each cell line. RNA-seq library was generated using TruSeq Stranded mRNA Sample Prep Kit (Illumina) and sequenced on Illumina HiSeq2500 by 75-bp paired-end sequencing. Raw RNA-seq read data for mouse ESC (GSE44067, ref.(Zhang et al., 2013)) and HT-29 (GSE41586, ref.(Xu et al., 2013)) were obtained from Gene Expression Omnibus. HT-1080 raw data (ENCSR535VTR) were obtained from ENCODE. The data were analysed using Kallisto(Bray et al., 2015) with the human RefSeq transcriptome as a reference. Transcripts per million reads were first calculated and then converted into fragments per kilo bases per million reads. Transcripts having the same gene symbol were merged and then a mean value for each gene was calculated.

For expression analysis of MB-3 treated MOLM-13, total RNA was purified from cells treated for 24h with 200 µM MB-3 with Trizol according to the manufacturer's instructions. Two independent extractions were performed. RNA-seq library was generated using Nextera library preparation kit and sequenced on Illumina 2500 by 100-bp paired end sequencing. Reads were mapped to Hg19 GRChg37 using GSNAP. Read counts were

obtained with HTSeq. Differential gene expression analysis was performed using DESeq2 and differentials called at a p-value<0.01 with a fold change of 1.5.

Generation of Cas9-expressing mouse line

The linearized Rosa26 targeting vector (25 µg) was electroporated into 10×10^6 JM8.F6 mouse ESCs at 230V and 500 mF using GenePluser II (BioRad) and the cells were plated onto three 10-cm dishes. Sixteen hours post electroporation, G418 selection was initiated at 180 µg ml^{-1}. G418-resistant colonies were picked 7 days later and homologous recombination was analysed by PCR using primer 5'-TCGCATTGTCTGAGTAGGTGTCATTCTA-3' and 5'-CTAACAAAACGTCTCAACTTCAAGGTGA-3' with LongAmp Taq DNA polymerase (NEB). Positive clones were further expanded and Cas9 function was assessed by the Cas9 reporter virus as described above. One targeted clone was injected into albino-B6 blastocysts (C57Bl/6-$Tyr^{c\text{-}Brd/c\text{-}Brd}$) and chimera males with high coat colour chimerism (>90%) were crossed with albino-B6 females to test germline transmission. Genotyping was carried out using HotStarTaq DNA polymerase (Qiagen) with primers: 5'-CTCTCCCAAAGTCGCTCTGA-3', 5'-GAAAGACCGCGAAGAGTTTGTC-3' and 5'-ACCCCAGATGACTACCTATCCT-3', yielding a 317-bp band from the Cas9 allele and a 395-bp band from the wild-type allele. The offspring of this crossing were used for lone-term survival assay and hematological analysis. The chimeras with germ line transmission were then crossed with C57Bl/6N females and inbred Cas9-expressing mouse line was established and maintained. All animal studies were carried out in accordance with the Animals (Scientific Procedures) Act 1986, UK and approved by the Ethics Committee at the Sanger Institute.

Isolation of mouse haematopoietic progenitors

$Flt3^{ITD/+}$ mice (Lee et al., 2007) were kindly provided by Gary Gilliland and crossed with $Rosa26^{Cas9/+}$ mice. Freshly isolated bone marrow from 6- to 10-week-old female wild-type, $Rosa26^{Cas9/+}$ or $Flt3^{ITD/+}$; $Rosa26^{Cas9/+}$ mice were used. Bone marrow cells were exposed to erythrocyte lysis (BD PharmLyse, BD Bioscience), followed by magnetic bead selection of Lin^- cells using the Lineage Cell Depletion Kit (Miltenyi Biotec, cat. no. 130-090-858) according to the manufacturer's instructions. Lin^- were cultured in X-VIVO 20 (Lonza) supplemented with 5% BIT serum (Stem Cell Technologies) 10ng ml^{-1} IL3 (Peprotech), 10ng ml^{-1} IL6 (Peprotech) and 50ng ml^{-1} of SCF (Peprotech).

Freshly dissected bone marrow cells (as mentioned above) were blocked with anti-mouse CD16/32 (BD Pharmigen, cat. no. 553142) and 10% mouse serum (Sigma). For the identification of LK/LSK subpopulations, staining was performed using CD4 PE/Cy5 (Biolegend, cat. no. 100514), CD5 PE/Cy5 (Biolegend, cat. no. 100610), CD8a PE/Cy5 (Biolegend, cat. no. 100710), CD11b PE/Cy5 (Biolegend, cat. no. 101210), B220 PE/Cy5 (Biolegend, cat. no. 103210), TER-119 PE/Cy5 (Biolegend, cat. no. 116210), GR-1 PE/Cy5 (Biolegend, cat. no. 108410), SCA-1 Pacific Blue (Biolegend, cat. no. 122520) and CD117 APC-eFluor780(eBioscience, cat. no. 47-1171). Flow cytometry analysis was performed using a LSRFortessa instrument (BD) and resulting data were subsequently analyzed using FlowJo.

For replating assays, 50,000 bone marrow cells from 3× WT and 3× $Rosa26^{Cas9/Cas9}$ mice were plated in M3434 (Stem Cell Technologies) and counted after 7 days with 30,000 cells replated, until 3rd replating.

Retrovirus production and transduction

Retrovirus constructs pMSCV-MLL-AF9-IRES-YFP(Dawson et al., 2011), pMSCV-MLL-AF4-PGK-puro(Montes et al., 2011) and package plasmid psi-Eco were used to produce retrovirus. 293T cells (Life Technologies) were cultured and prepared for transduction in 10cm plates as described above. For virus production, 5 µg of the above plasmids and 5 µg psi-Eco packaging vector were transfected drop wise into the 293T cells using 47.5 µl TransIT LT1 (Mirus) and 600 µl Opti-MEM (Invitrogen). The resulting viral supernatant was harvested as previously described. Transduction of primary mouse cells was performed in 6-well plates as mentioned above.

After transduction, YFP positive cells were sorted for MLL-AF9 and puromycin resistant cells selected (1.5 µg ml^{-1} concentration) for MLL-AF4.

Whole-body bioluminescent imaging

For *in vivo* experiments, MOLM-13 cells expressing Cas9 were first transduced with a firefly luciferase–expressing plasmid (System Biosciences). After propagation, the cells were transduced with a lentivirus expressing either empty or KAT2A gRNA (day 0) and selected with puromycin from day 2 to day 5. At day 5 post transduction, the cells were suspended in fresh medium without puromycin. At day 7, 1 x 10^5 cells were transplanted into a *Rag2*$^{-/-}$ *IL2RG*$^{-/-}$ mouse by tail-vein injection. At day 17 post-transplant, the tumor burdens of the animals were detected using IVIS Lumina II (Caliper) with Living Image version 4.3.1 software (PerkinElmer). Briefly, 100 µl of 30 mg/ml D-luciferin (BioVision) was injected into the animals intraperitoneally. Ten min after injection, the animals were maintained in general anesthesia by isoflurane and put into the IVIS chamber for imaging. The detected tumor burdens were measured and quantified by the same software. The animals were culled when the tumor burden was 10^9 photons per second or higher. All animal studies were carried out in accordance with the Animals (Scientific Procedures) Act 1986, UK and approved by the Ethics Committee at the Sanger Institute. Randomisation and blinding were not applied.

Western blot anlaysis

MOLM-13 was transduced with a lentivirus expressing the KAT2A gRNA(1) or an empty control and selected with 1.0 µg ml^{-1} puromycin for 3 days starting from day 2 post transduction. The cells were further cultured for 2 days and then lysed. The lysates were used for SDS-PAGE. Anti-KAT2A (Santa Cruz Biotech, cat. no. sc-20698) and ACTB (Abcam, ab8227) were used for immunoblot analysis.

Chromatin immunoprecipitation and quantitative PCR (ChIP-qPCR) analysis

ChIP was performed as described (Fong et al., 2015) with minor modifications. MOLM-13 was treated for 24h with either DMSO (0.1%, vehicle) or MB-3 (100 µM). Cross-linked cell pellets were snap-frozen, kept at -80 °C and thawed immediately prior to lysis and sonication. Antibody incubation times were 5.5h to overnight. Experiments were performed as paired biological duplicates, with single cultures split for treatment in each replicate experiment. Antibodies used for immunoprecipitation (IP) were anti-H3K27ac (Abcam, cat. no. ab4729) and anti-H3K9ac (Abcam, cat. no. ab10812). 1x 10^7 cells were used in each IP. Primers used for qPCR analysis were designed against evolutionary conserved regions (ECR Browser, https://ecrbrowser.dcode.org/); MEIS1-F, CCAGAAGAAGACAGAGCGGA; MEIS1-R, CCCTCAGACCCAACTACCAA; HOXA10-F, GTTTATAGCGGCGCATTCCA; HOXA10-R, CGGGTTTGATTTCTGAGCCC; HOXA9-F, CGCTCTCATTCTCAGCATTG; HOXA9-R, TTAAACCTGAACCGCTGTCG; MYC-F, CACTCTCCCTGGGACTCTTG; MYC-R, TCTCCCTTTCTCTGCTGCTC; GAPDH-e3-F, CAAATTCCATGGCACCGTCA; GAPDH-e3-R, TCCTGGAAGATGGTGATGGG. qPCR reactions were performed using Brilliant II SYBR Green QPCR Master Mix (Agilent Technologies) in a CFX96 RealTime System (BioRad).

May-Grunwald-Giemsa cytospin staining

10^5 cells were cytospun for 5 min at 300*g* onto glass slides. Slides were then stained for 3 min with May-Grunwald solution (Sigma-Aldrich) at room temperature. After washing in water, they were incubated for 20 min in Giemsa solution (Sigma-Aldrich) (1:20 in water). Slides were washed again in water before being mounted with Mowiol embedding medium.

Flow cytometry analyses of MB-3 treated AML cells

Cells were treated for 24 h with 100 µM MB-3, stained with CD13 (clone WM-15; eBioscience). Apoptosis levels were measured in AML cell lines treated with 100 µM MB-3 for 24, 72 and 144 h, respectively, by using Annexin V (Life Technologies, cat. no. V13242). Data were analysed by using LSRFortessa (BD) or Gallios (Beckman Coulter) instruments.

Analysis of nucleotide biases on CRISPR dropout efficiency

Raw read counts of gRNAs from mouse ESC day 14 samples and the matched plasmid were first normalised by total number of reads and then fold change was calculated for each gRNA. Genes that had 3 or more gRNAs with ≥ 4-fold reduction were extracted as depleted genes. Amongst gRNAs targeting these genes, gRNAs whose fold reduction was less than or more than 4 were grouped as "inefficient" or "efficient" gRNAs, respectively. For each position, four 2x2 contingency tables with each base vs the rest in the columns and with the numbers of inefficient and efficient gRNAs in the rows were generated and the X^2 test was performed. When a fraction of a given base in the inefficient gRNAs is greater or smaller than that in the efficient gRNAs, the base is considered as disfavoured or favoured, respectively.

Time-course depletion analysis in HT-29

The dendrogram was obtained by performing a hierarchical clustering of the depletion signals of genes that were significantly depleted (FDR < 10%) at day 25, across time points by using the average linkage method and the Euclidean distance as metric. The cutting threshold of the dendrogram was selected heuristically as a trade-off between the number of resulting clusters and their silhouette widths. For each of the resulting 7 clusters (composition reported in Table S2, 1st sheet), a plot containing all the included depletion signals as well as the depletion signal of the centroid (together with its standard deviation) was generated as shown in Figure 2D. For each of the centroid signal, a plateau time was defined as the minimal time point *t* such that there are no other time points for which the $-\log_{10}$ (depletion *P* values) of the centroid signal exceeds that reached at *t* plus half of its standard deviation. Based on the proximities of their plateau time the clusters were then grouped into three classes: Early (plateau ≤ day 10), Intermediate (plateau ≤ day 16) and Late (plateau = day 22). Genes whose signal belongs to one of these classes were pooled together and a gene ontology term and KEGG pathway enrichment analysis was performed. Results of this analysis are reported in Table S2 (2nd to 4th sheets) and representative terms and pathways were shown in Figure 2E.

Quality control analysis of CRISPR screens by GSEA

As a quality control assessment of each dropout screen, we conducted gene set enrichment analysis (GSEA) on the following 9 signatures from the MSigDB portal (http://www.broadinstitute.org/gsea/msigdb/index.jsp): DNA_REPLICATION, KEGG_PROTEASOME, KEGG_RNA-POLYMERASE, KEGG_SPLICEOSOME, PROTEASOME_COMPLEX, REACTOME_DNA_REPLICATION, RibosomalProteins_lit, RNA_POLYMERASE_COMPLEX, and SPLICEOSOME. For each cell line, we ranked genes based on a Depletion/Enrichment (D/E) score given by the sum of the $\log_{10}$(depletion *P* value) and the negative $\log_{10}$(enrichment *P* value), and run the GSEA tool(Subramanian et al., 2005) over them using the collected signatures as queries. The significance of the obtained enrichment scores were computed as nominal *P* values from permutation tests (1,000 trials with randomly generated signatures of the same sizes of the real ones). Results were found in Supplementary Data 2. Selected results were shown in Figures S3E and S5L.

Pathway enrichment analysis

Gene ontology terms, KEGG and REACTOME pathways enriched in the "pan-essential" genes were analysed using the MSigDB website (http://www.broadinstitute.org/gsea/msigdb/index.jsp). Gene ontologies enriched in the AML-specific essential genes were analysed using the DAVID website (https://david.ncifcrf.gov/).

Statistical analysis

Statistical analyses performed were specified in figure legends. Differences were considered significant for *P* values < 0.05.

References

Bray, N.L., Pimentel, H., Melsted, P., and Pachter, L. (2015). Near-optimal RNA-Seq quantification. arXiv preprint *rXiv:1505.02710*.

Chen, B., Gilbert, L.A., Cimini, B.A., Schnitzbauer, J., Zhang, W., Li, G.W., Park, J., Blackburn, E.H., Weissman, J.S., Qi, L.S.*, et al.* (2013). Dynamic imaging of genomic loci in living human cells by an optimized CRISPR/Cas system. Cell *155*, 1479-1491.

Cong, L., Ran, F.A., Cox, D., Lin, S., Barretto, R., Habib, N., Hsu, P.D., Wu, X., Jiang, W., Marraffini, L.A.*, et al.* (2013). Multiplex genome engineering using CRISPR/Cas systems. Science *339*, 819-823.

Dawson, M.A., Prinjha, R.K., Dittmann, A., Giotopoulos, G., Bantscheff, M., Chan, W.I., Robson, S.C., Chung, C.W., Hopf, C., Savitski, M.M.*, et al.* (2011). Inhibition of BET recruitment to chromatin as an effective treatment for MLL-fusion leukaemia. Nature *478*, 529-533.

Fong, C.Y., Gilan, O., Lam, E.Y.N., Rubin, A.F., Ftouni, S., Tyler, D., Stanley, K., Sinha, D., Yeh, P., Morison, J.*, et al.* (2015). BET inhibitor resistance emerges from leukaemia stem cells. Nature *525*, 538-542.

Koike-Yusa, H., Li, Y., Tan, E.P., Velasco-Herrera Mdel, C., and Yusa, K. (2014). Genome-wide recessive genetic screening in mammalian cells with a lentiviral CRISPR-guide RNA library. Nature biotechnology *32*, 267-273.

Lee, B.H., Tothova, Z., Levine, R.L., Anderson, K., Buza-Vidas, N., Cullen, D.E., McDowell, E.P., Adelsperger, J., Frohling, S., Huntly, B.J.*, et al.* (2007). FLT3 mutations confer enhanced proliferation and survival properties to multipotent progenitors in a murine model of chronic myelomonocytic leukemia. Cancer cell *12*, 367-380.

Li, W., Xu, H., Xiao, T., Cong, L., Love, M.I., Zhang, F., Irizarry, R.A., Liu, J.S., Brown, M., and Liu, X.S. (2014). MAGeCK enables robust identification of essential genes from genome-scale CRISPR/Cas9 knockout screens. Genome biology *15*, 554.

Mali, P., Yang, L., Esvelt, K.M., Aach, J., Guell, M., DiCarlo, J.E., Norville, J.E., and Church, G.M. (2013). RNA-guided human genome engineering via Cas9. Science *339*, 823-826.

Montes, R., Ayllon, V., Gutierrez-Aranda, I., Prat, I., Hernandez-Lamas, M.C., Ponce, L., Bresolin, S., Te Kronnie, G., Greaves, M., Bueno, C.*, et al.* (2011). Enforced expression of MLL-AF4 fusion in cord blood CD34+ cells enhances the hematopoietic repopulating cell function and clonogenic potential but is not sufficient to initiate leukemia. Blood *117*, 4746-4758.

Pettitt, S.J., Liang, Q., Rairdan, X.Y., Moran, J.L., Prosser, H.M., Beier, D.R., Lloyd, K.C., Bradley, A., and Skarnes, W.C. (2009). Agouti C57BL/6N embryonic stem cells for mouse genetic resources. Nature methods *6*, 493-495.

Subramanian, A., Tamayo, P., Mootha, V.K., Mukherjee, S., Ebert, B.L., Gillette, M.A., Paulovich, A., Pomeroy, S.L., Golub, T.R., Lander, E.S.*, et al.* (2005). Gene set enrichment analysis: a knowledge-based approach for interpreting genome-wide expression profiles. Proceedings of the National Academy of Sciences of the United States of America *102*, 15545-15550.

Xu, X., Zhang, Y., Williams, J., Antoniou, E., McCombie, W.R., Wu, S., Zhu, W., Davidson, N.O., Denoya, P., and Li, E. (2013). Parallel comparison of Illumina RNA-Seq and Affymetrix microarray platforms on transcriptomic profiles generated from 5-aza-deoxy-cytidine treated HT-29 colon cancer cells and simulated datasets. BMC bioinformatics *14 Suppl 9*, S1.

Zhang, Y., Wong, C.H., Birnbaum, R.Y., Li, G., Favaro, R., Ngan, C.Y., Lim, J., Tai, E., Poh, H.M., Wong, E.*, et al.* (2013). Chromatin connectivity maps reveal dynamic promoter-enhancer long-range associations. Nature *504*, 306-310.
