# Supplementary figures and images for: A CRISPR Dropout Screen Identifies Genetic Vulnerabilities and Therapeutic Targets in Acute Myeloid Leukemia
Source: Cell Rep. 2016 Oct 18;17(4):1193–205. doi: 10.1016/j.celrep.2016.09.079 (PMC5081405; doi:10.1016/j.celrep.2016.09.079)

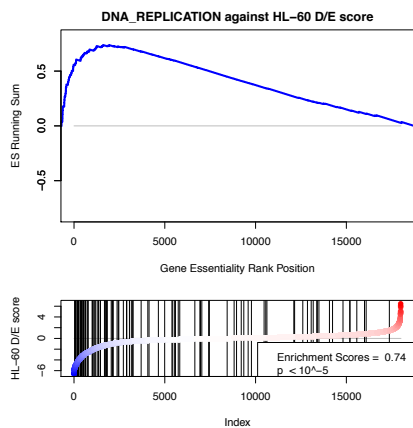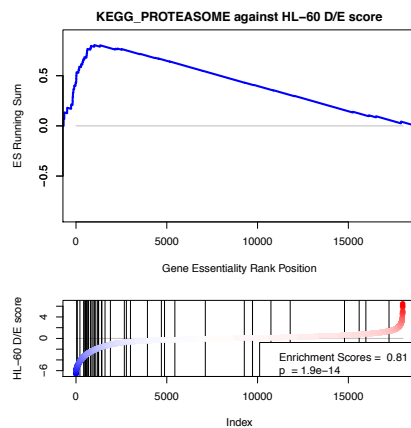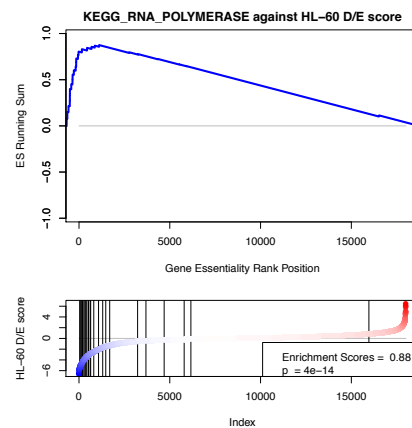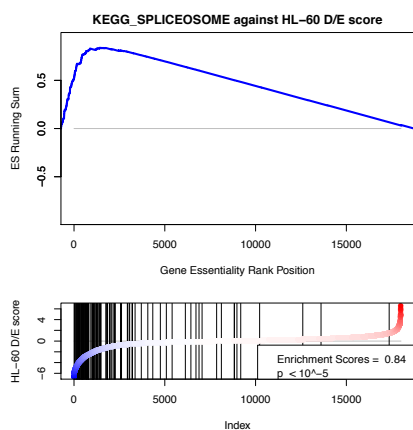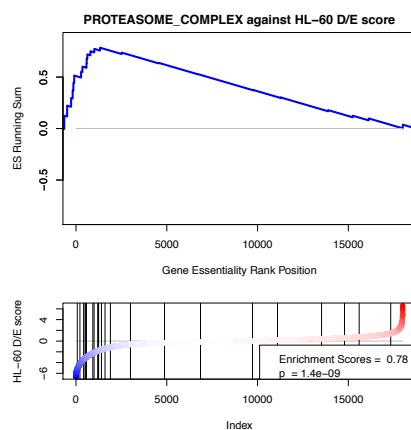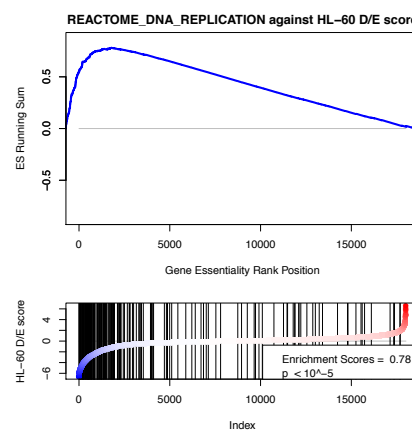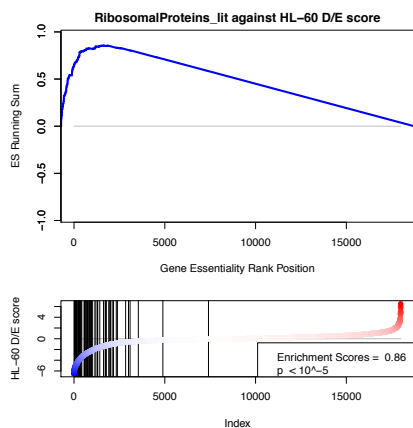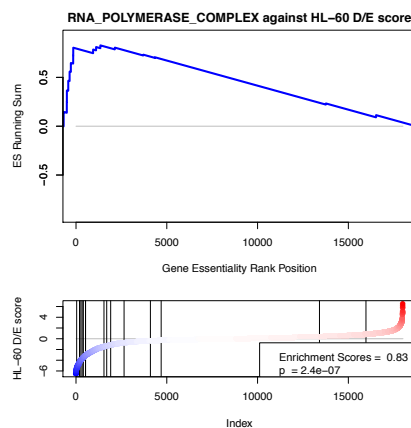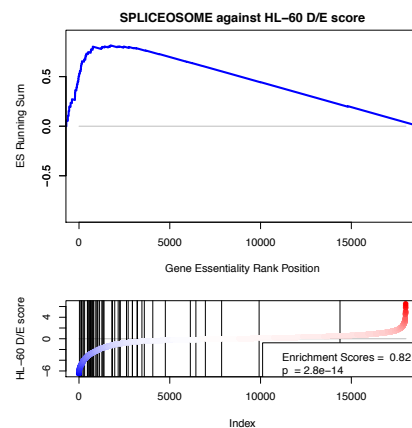

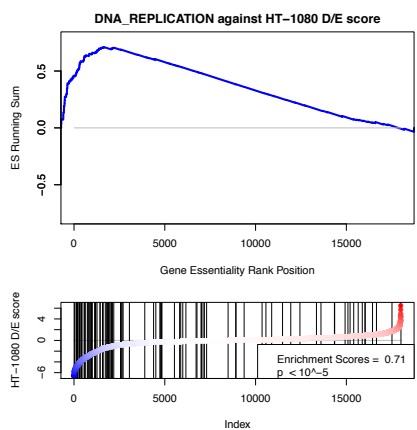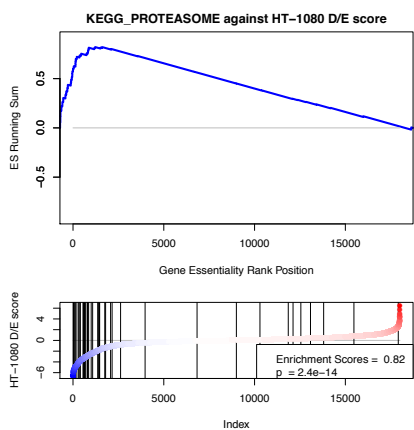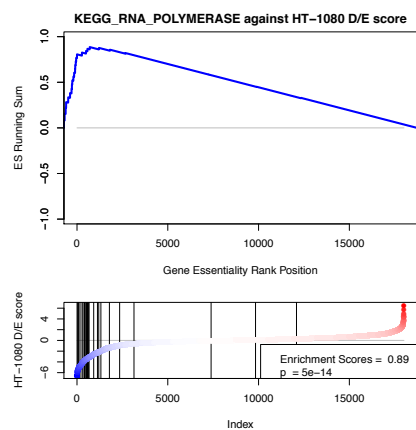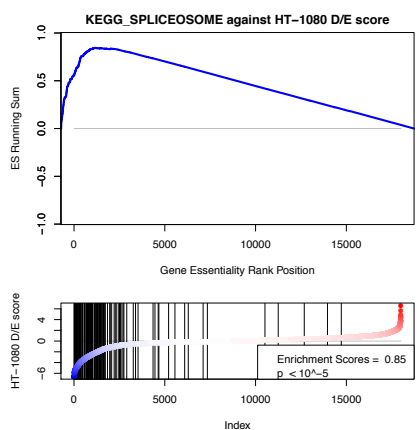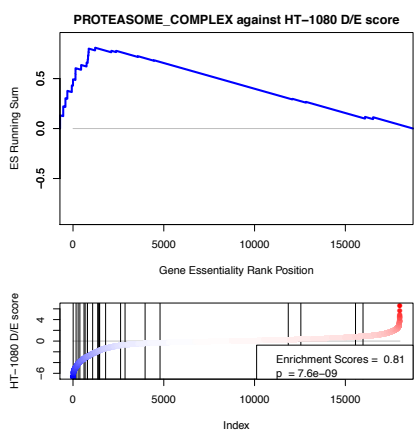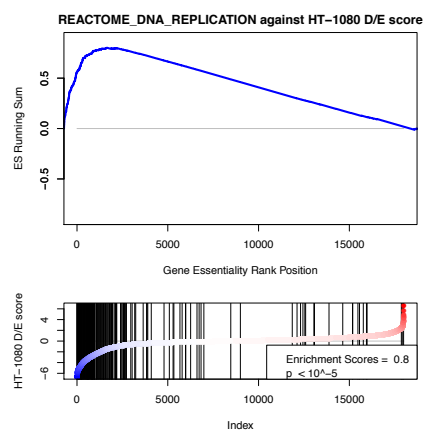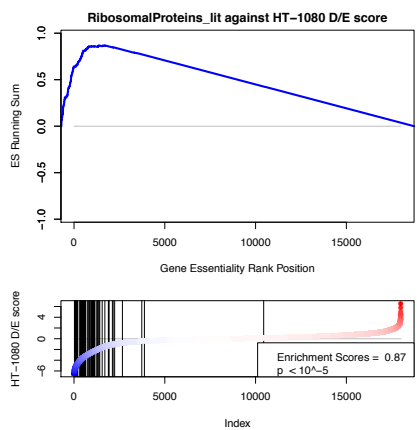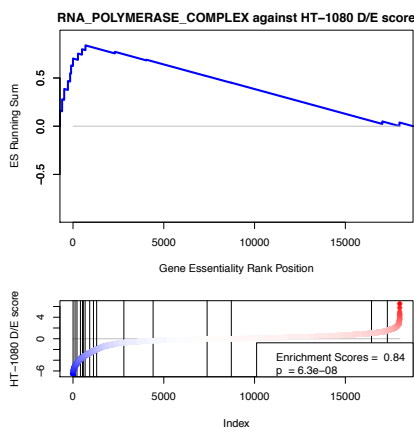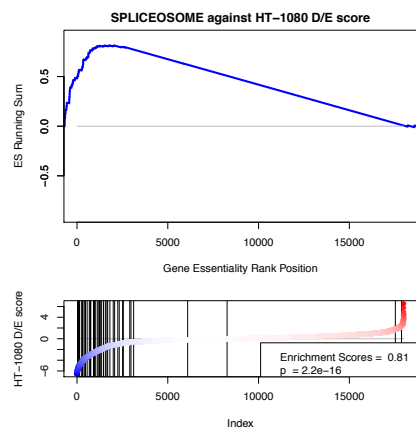

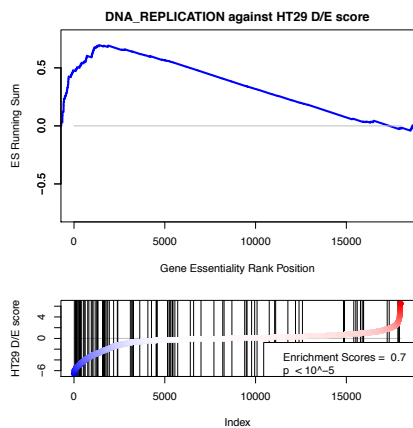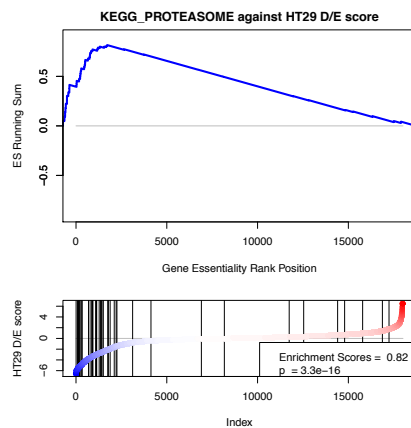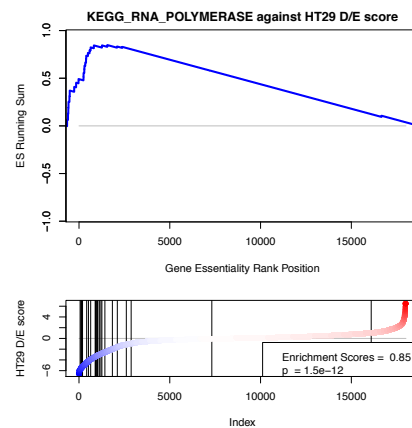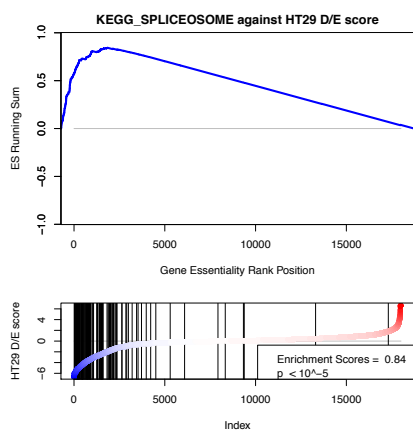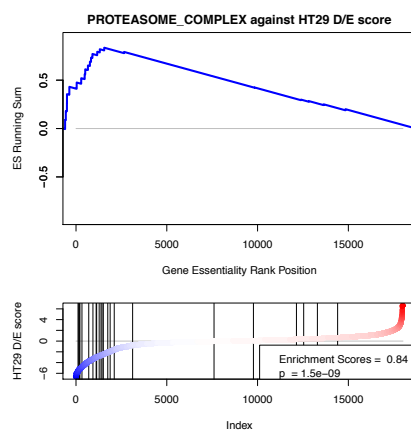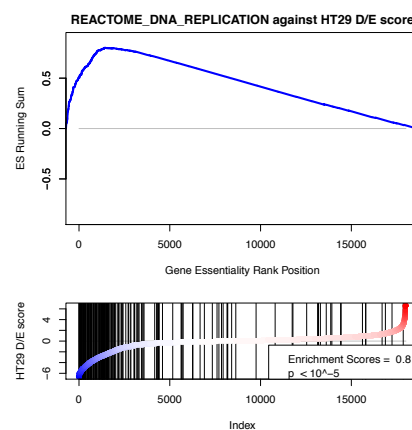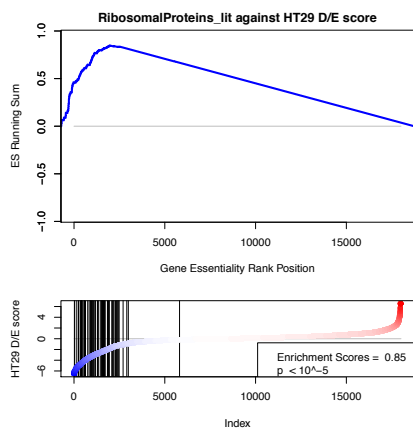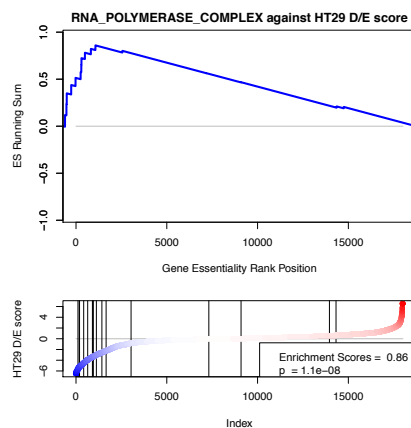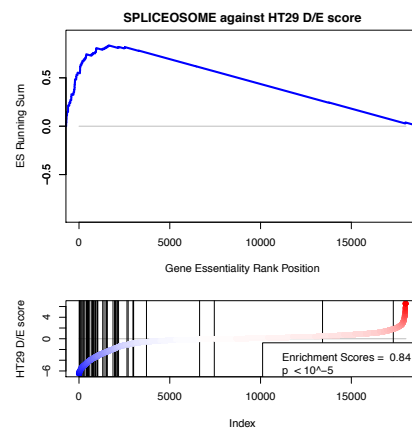

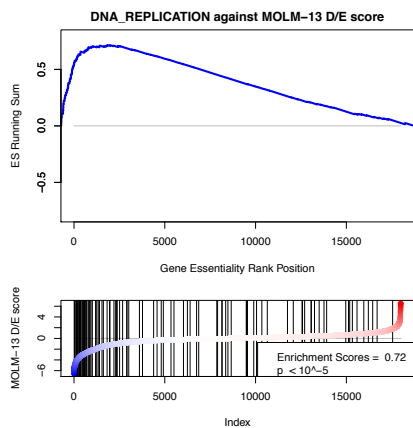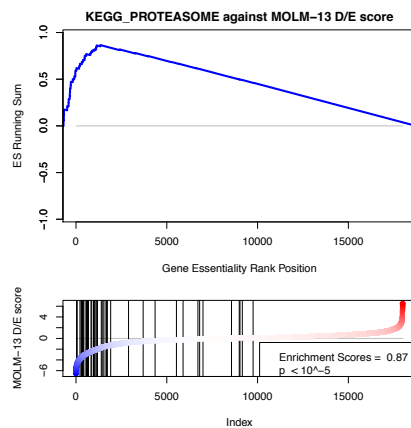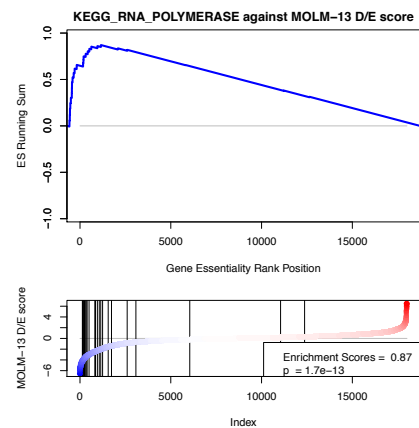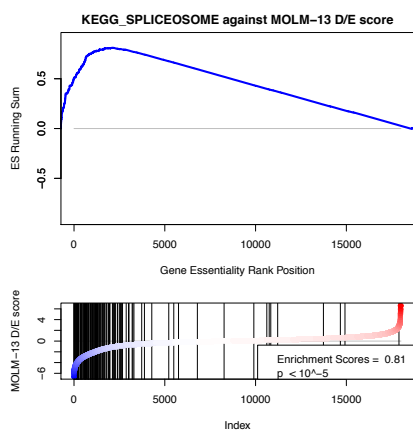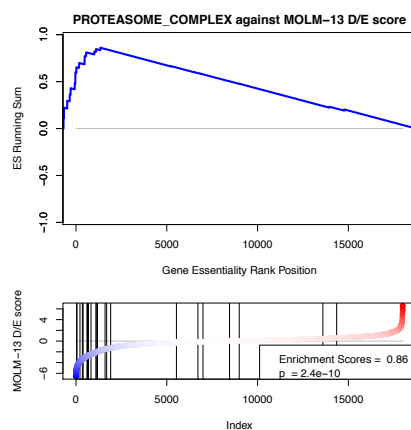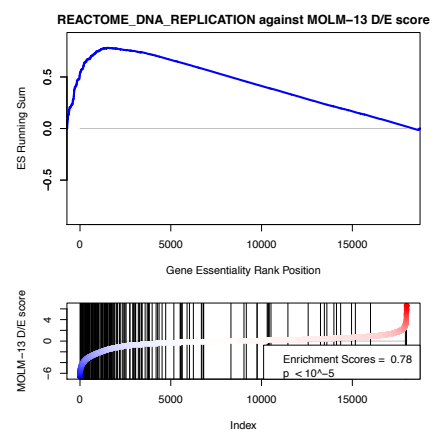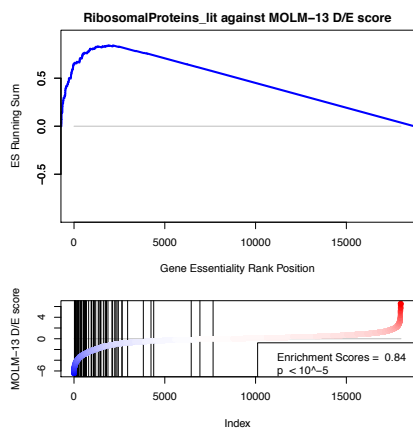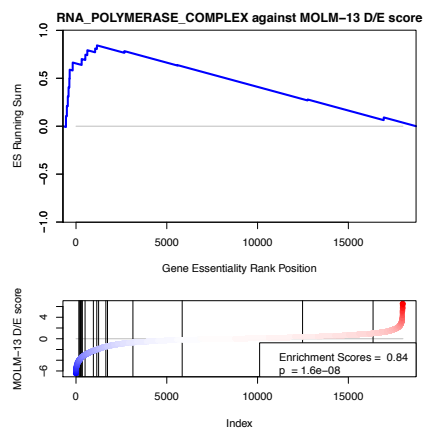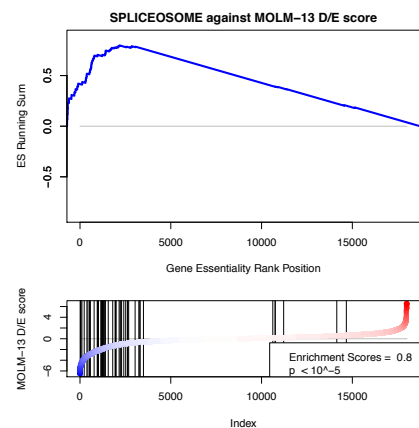

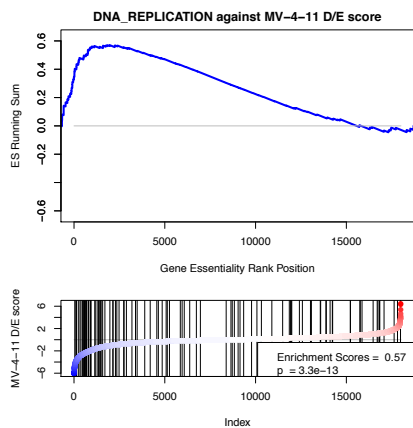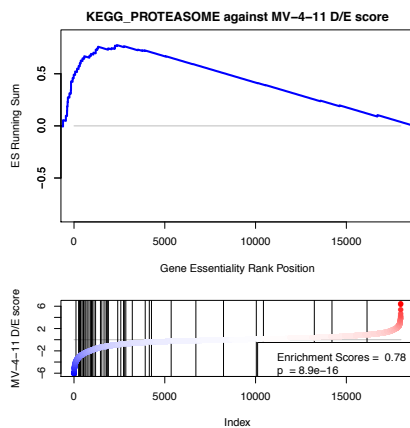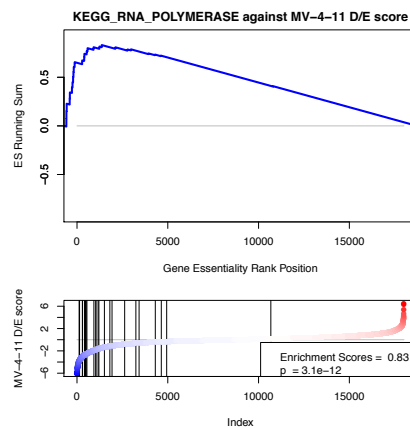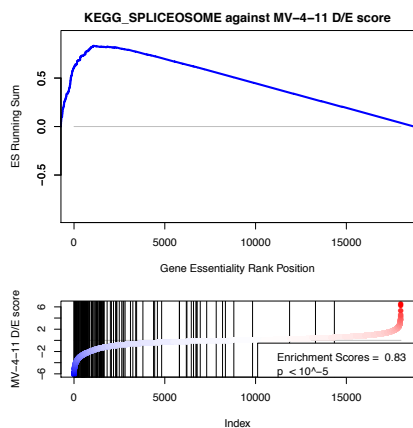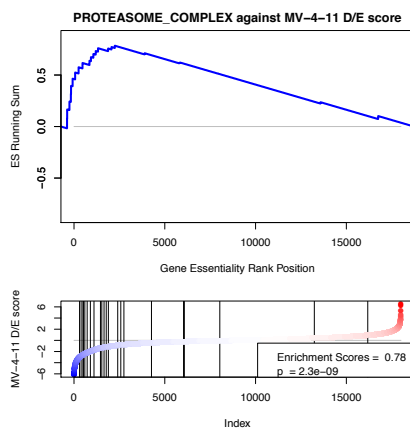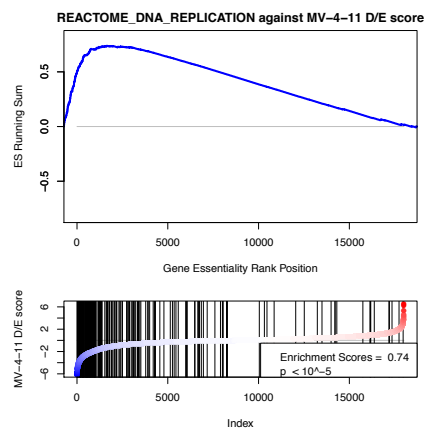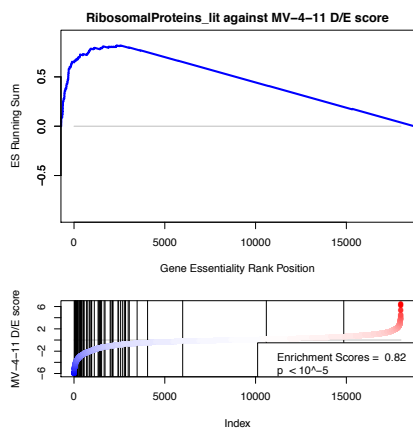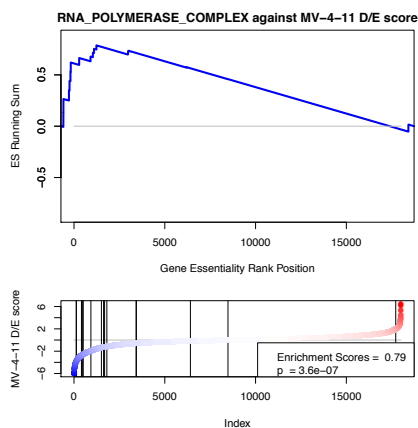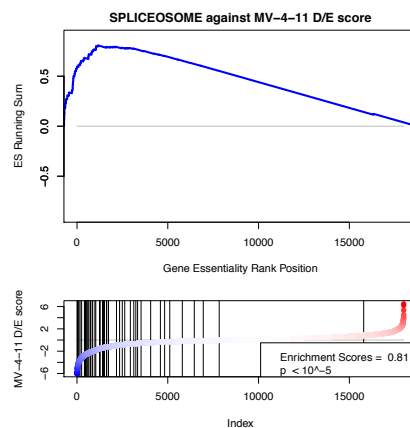

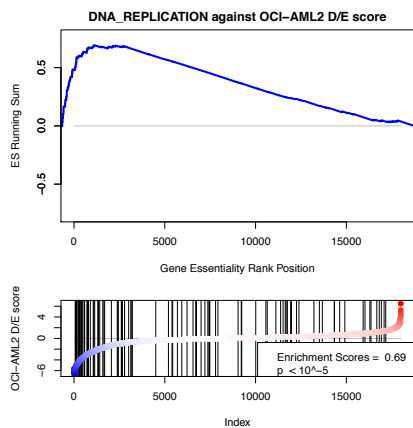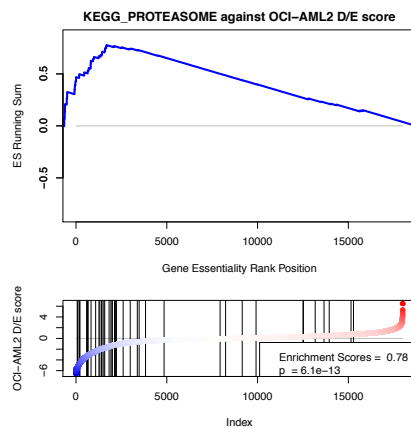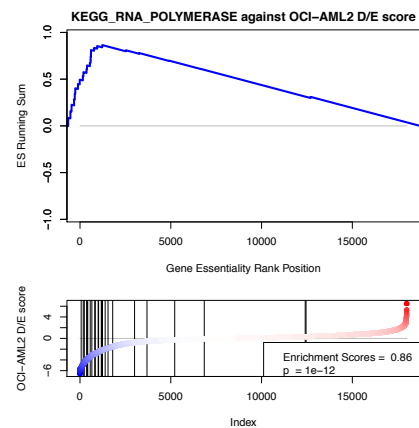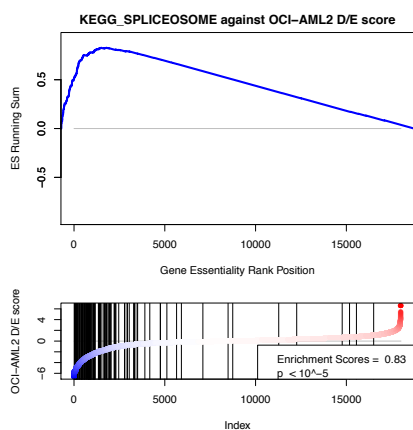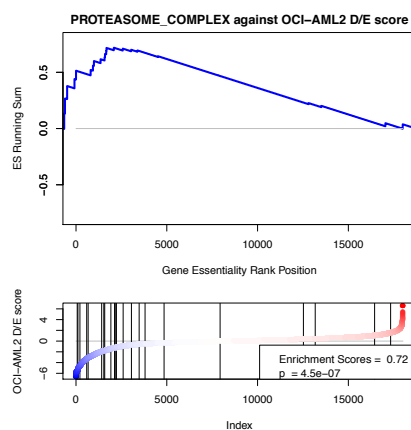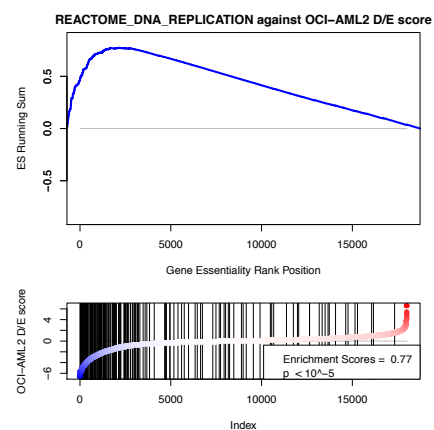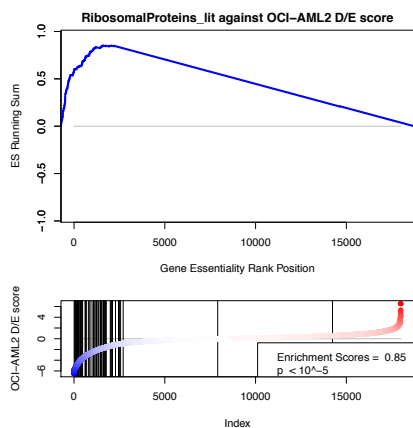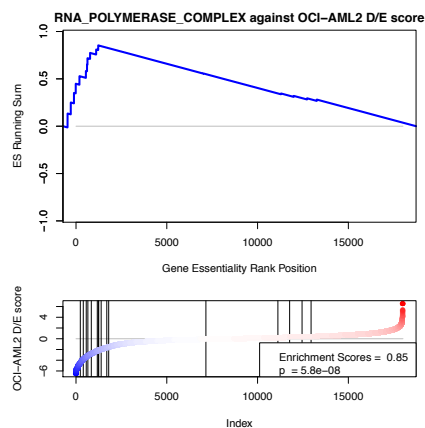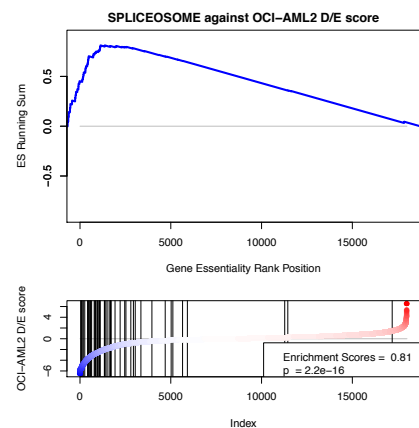

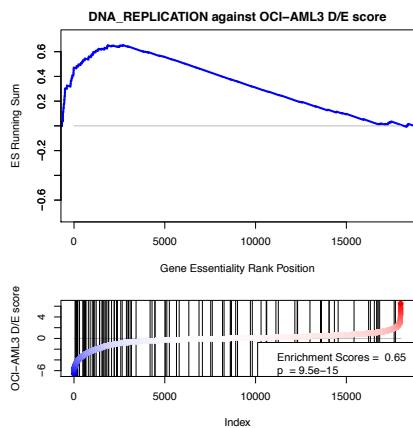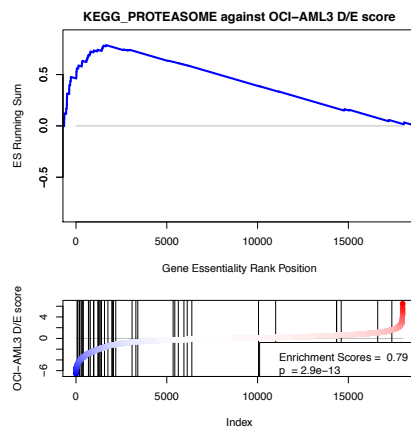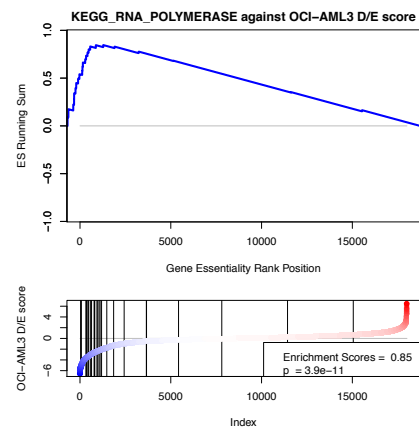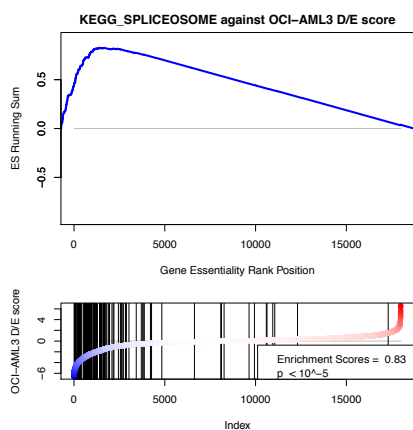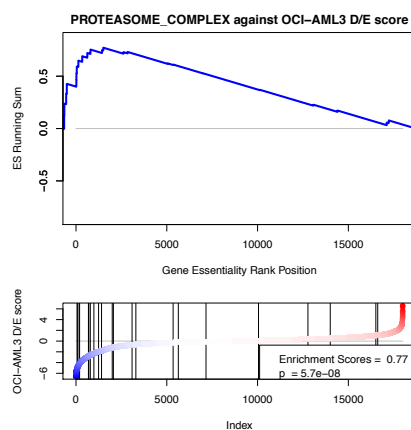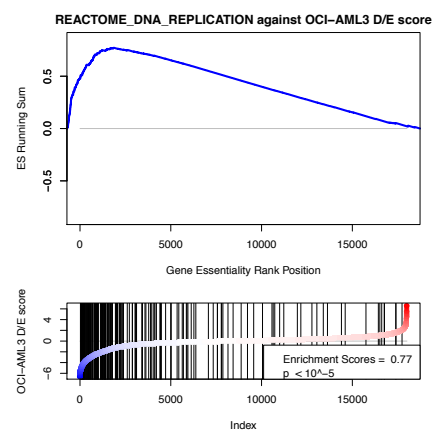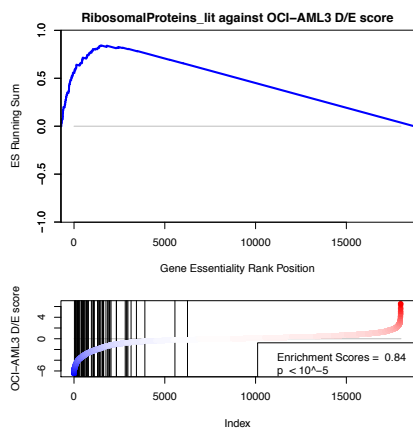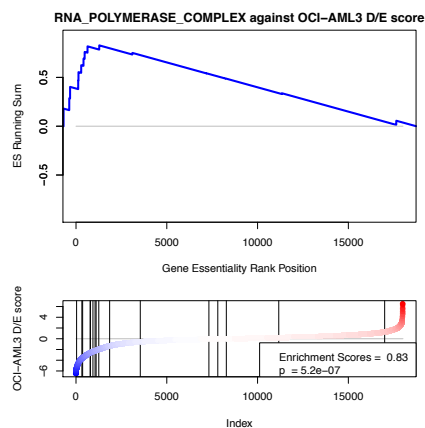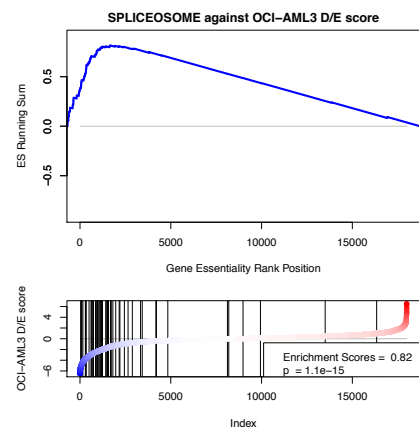

Supplement: Data S2. Human CRISPR Screen Data, Related to Figures 2 and 3 [file mmc6.zip › GSEA_QC_Results.pdf]
